# Dynamic modeling of Nrf2 pathway activation in liver cells after toxicant exposure

**DOI:** 10.1038/s41598-022-10857-x

**Published:** 2022-05-05

**Authors:** Steven Hiemstra, Mirjam Fehling-Kaschek, Isoude A. Kuijper, Luc J. M. Bischoff, Lukas S. Wijaya, Marcus Rosenblatt, Jeroen Esselink, Allard van Egmond, Jornt Mos, Joost B. Beltman, Jens Timmer, Bob van de Water, Daniel Kaschek

**Affiliations:** 1 Division of Drug Discovery and Safety (DDS), Leiden Academic Centre for Drug Research (LACDR), Leiden University, Leiden, The Netherlands; 2 Institute of Physics, University of Freiburg, 79104 Freiburg, Germany; 3 Freiburg Center for Data Analysis and Modeling (FDM), University of Freiburg, 79104 Freiburg, Germany; 4 Centre for Integrative Biological Signalling Studies (CIBSS), University of Freiburg, 79104 Freiburg, Germany; 5 IntiQuan GmbH, Basel, Switzerland

**Keywords:** Computer modelling, Systems analysis, Software

## Abstract

Cells are exposed to oxidative stress and reactive metabolites every day. The Nrf2 signaling pathway responds to oxidative stress by upregulation of antioxidants like glutathione (GSH) to compensate the stress insult and re-establish homeostasis. Although mechanisms describing the interaction between the key pathway constituents Nrf2, Keap1 and p62 are widely reviewed and discussed in literature, quantitative dynamic models bringing together these mechanisms with time-resolved data are limited. Here, we present an ordinary differential equation (ODE) based dynamic model to describe the dynamic response of Nrf2, Keap1, Srxn1 and GSH to oxidative stress caused by the soft-electrophile diethyl maleate (DEM). The time-resolved data obtained by single-cell confocal microscopy of green fluorescent protein (GFP) reporters and qPCR of the Nrf2 pathway components complemented with siRNA knock down experiments, is accurately described by the calibrated mathematical model. We show that the quantitative model can describe the activation of the Nrf2 pathway by compounds with a different mechanism of activation, including drugs which are known for their ability to cause drug induced liver-injury (DILI) i.e., diclofenac (DCF) and omeprazole (OMZ). Finally, we show that our model can reveal differences in the processes leading to altered activation dynamics amongst DILI inducing drugs.

## Introduction

Partial reduction of oxygen molecules leads to formation of reactive oxygen species (ROS) or reactive metabolites (RM). When ROS or RM accumulate within a cell, oxidative stress occurs. Cells have evolved a protective anti-oxidant defense mechanism to prevent oxidative stress from damaging the cell. One important defense mechanism is the tripeptide glutathione (GSH), which is able to absorb and inactivate ROS and RM. Moreover, activation of a protective signaling pathway is enabled by nuclear translocation of a key transcription factor, nuclear factor erythroid 2-like 2 (NFE2L2/Nrf2)^1^. Under basal conditions, Nrf2 is bound by Kelch-like ECH-associated protein 1 (KEAP1/Keap1), which targets Nrf2 for ubiquitination and subsequent proteasomal degradation^1^. Upon oxidative stress, ROS or RM can bind to cysteine residues on the Keap1 protein, which alters the conformation of the Keap1–Nrf2 complex and thereby avoids ubiquitination and degradation of Nrf2^2,3^. Newly assembled Nrf2 proteins therefore accumulate in the cytoplasm and subsequently translocate to the nucleus. Within the nucleus, Nrf2 binds to the antioxidant-response element (ARE) which promotes transcription of hundreds of target genes such as the detoxifying enzymes heme oxygenase (Hmox1), NAD(P)H dehydrogenase 1 (Nqo1) and sulfiredoxin 1 (Srxn1), xenobiotic metabolizing enzymes and genes involved in glutathione synthesis, i.e., glutathione reductase (GSR) and glutamate-cysteine ligase modifier (GCLM)^4–7^.

Nrf2 activation has been linked to clinical pathological outcomes^8^ and occurs during exposure to toxicants that have liabilities for target organ toxicities e.g. drug-induced liver injury (DILI)^9,10^. DILI is a major concern both in the clinic and on the market. Drugs account for 50% of all liver failures in the United States^11^. Furthermore, DILI is the most important cause for drug withdrawal from the market^12^. Drug-induced cell injury leads to the activation of cellular stress response pathways^13–15^. These pathways are typically involved in repair of cell injury and restoration of cellular homeostasis. Alternatively, the stress response pathways may initiate progression to cellular demise. The balance between adaptive response versus progression injury is likely dependent on the amplitude of cell injury and consequently the amplitude of pathway activation. Monitoring of the quantitative aspects of adaptive stress response pathway activation is pivotal to understand and compute the relationships between pathway activity and cellular demise. The Nrf2 pathway is one of the major adaptive stress response pathways involved in DILI. It is known to be upregulated by various drugs inducing liver injury^9,15^. Furthermore, *in vivo* data show that Nrf2 has an essential role in protection against hepatotoxicants, as Nrf2 knock-out mice have a higher susceptibility to a variety of hepatotoxicants compared to wild-type mice^10^.

To study adaptive stress response pathways in more detail, we employ quantitative dynamic modeling. This type of modeling aims to quantitatively describe molecular processes involved in these stress responses, leading to more detailed mechanistic understanding of these pathways^16^. Ultimately, quantitative dynamic modeling could aid in a fully mechanistic understanding of DILI. Although Nrf2 signaling is of pivotal importance in cellular defense, only a few models are available. The first Nrf2 model was published by Zhang et al.^17^, mainly focusing on the steady-state dose response relationship between the stress-inducing compound and different constituents of the Nrf2 pathway. Together with follow-up publications based on this initial model, this modeling effort resulted in an extended and detailed pathway representation based on cyclosporine A exposure to renal cells^17–20^. More recently, the same group adapted the model to acetaminophen induction of Nrf2 signaling in liver cells^21^. To date none of the existing Nrf2 models have studied the Nrf2 activation mechanism. Therefore, we set out to establish quantitative dynamic models of the Nrf2 pathway based on ordinary differential equations of the Nrf2 signaling pathway. First, we established a dataset of different constituents of the Nrf2 signaling pathway using fluorescently labeled reporter cell lines (Keap1-GFP, Nrf2-GFP and Srxn1-GFP). With time-resolved high throughput confocal imaging we followed the dynamics of the individual components of the Nrf2 pathway after exposure to the soft-electrophile diethyl maleate (DEM) that targets Keap1^22^. In addition, mRNA levels, GSH measurements and knock-down experiments were applied to validate the Nrf2 model. Finally, we applied the model to diclofenac (DCF) and omeprazole (OMZ) to evaluate its application for compounds with DILI liabilities. Altogether, we established a quantitative Nrf2 model which is able to represent Nrf2 activation for both DEM, DCF and OMZ, thereby deepening our knowledge of the dynamics of Nrf2 signaling and Nrf2 activation after xenobiotic exposure.

## Results

### Constructing a model of Nrf2 pathway dynamics

**Figure 1:**
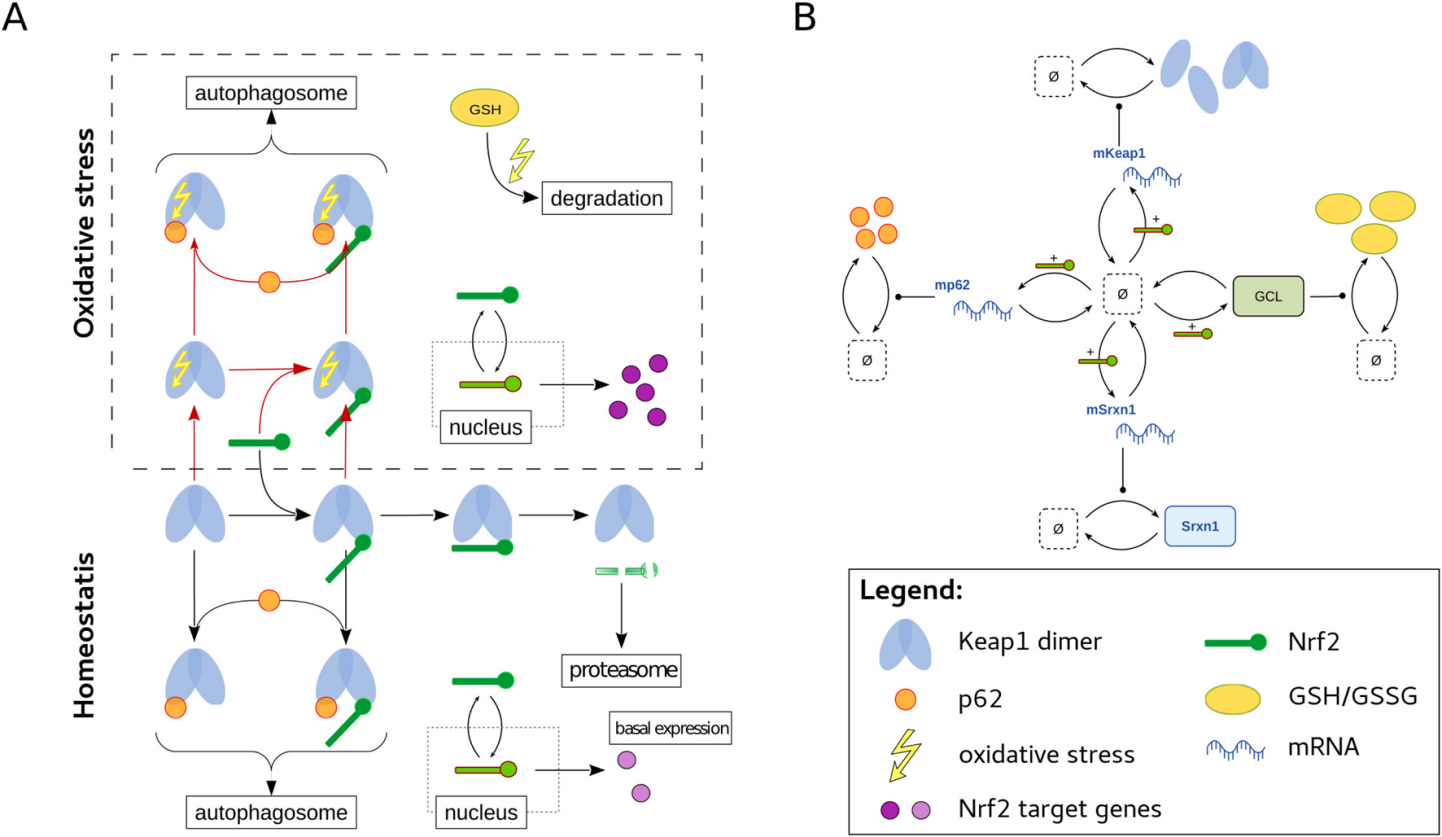
Pathway scheme. (**A**) Homodimers of Keap1 (shown in blue) are able to bind the ETGE (high affinity or hinge binding) and the DLG (low affinity or latch binding) motifs of Nrf2 (shown in green). When both Nrf2 motifs are bound, Keap1 can target Nrf2 for proteasomal degradation. p62 (shown in orange) is able to compete with Nrf2 for its latch binding to Keap1, resulting in autophagosomal degradation of Keap1. Upon oxidative stress (yellow arrow), Keap1 is no longer able to target Nrf2 for proteasomal degradation by lack of binding of the DLG motif. This results in nuclear accumulation of Nrf2 and target gene transcription including SRXN1, NQO1, HMOX1 and GLCM (shown in purple). In addition, oxidative stress is reduced via GSH (shown in yellow). (**B**) Scheme explaining the considered target gene expression of Nrf2 upon oxidative stress. Reactions between the constituents are represented by arrows. They are translated into differential equations for the quantitative model.

Our quantitative dynamic ODE model of the Nrf2 pathway focuses on the most important components of the Nrf2 signaling pathway (Fig. 1). Specifically, intracellular levels of Nrf2 are controlled by its negative regulator Keap1. Keap1 consists of a Broadcomplex–Tramtrack–Bric-a-Brac (BTB) domain, an intervening region (IVR), a double glycin repeat (DGR) and a C-terminal region (CTR). Keap1 forms a homodimer by dimerisation of BTB domains. The DGR and CTR domains of Keap1 (Keap1-DC) are able to bind two motifs present within the Nrf2 protein: the ETGE and the DLG motifs. Out of these two motifs, Keap1 has the highest affinity for the ETGE motif, forming the so-called hinge binding. Upon such binding, the other Keap1 molecule of the Keap1 dimer can bind the lower affinity DLG motif of Nrf2 (latch binding)^23,24^. When both the ETGE and the DLG motif are bound, Nrf2 will be poly-ubiquitinated and subsequently degraded by the proteasome. However, not only Nrf2 is able to bind to Keap1. Also p62 (*SQSTM1*/p62) is able to bind to the Keap1-DC region^24–26^. As a member of the autophagosome machinery, p62 will be recruited for autophagosomal degradation including its binding partners Keap1 and potentially Nrf2 (being attached to the second Keap1 dimer unit). After accumulation of RM or ROS, cysteine residues in the IVR can be modified introducing a conformational change in Keap1. This conformational change alters the binding pocket of Keap1-DC and prevents Nrf2-DLG to establish binding to Keap1 and thus ceasing Nrf2 degradation^23^. Newly synthesized Nrf2 translocates to the nucleus where it transcribes antioxidant response genes. Nrf2 target transcription also leads to upregulation of cellular glutathione (GSH) levels. Upon antioxidant binding GSH reduces disulfide bonds leading to formation of GSSG. The schematic pathway is interpreted mechanistically to derive a reaction network for the pathway components that is translated into an ODE model. The full set of reactions and corresponding equations are described in Supplement Section 1. Note that the resulting ODE model only includes the most important Nrf2-related components and does not take into account many additional components. For instance, the model does not include competitive binders for NRF2 to the ETGE motif on Keap1, such as WTX, p21, PALB and DPP3^27–30^, or other components that can affect Nrf2 directly, such as $\beta$-TRCP and Protein kinase C^31,32^. The ODE model makes quantitative predictions of the time-course of all dynamic states whether they are observed or not. A major goal of our modeling approach is to test if the mechanisms proposed in literature not only qualitatively but also quantitatively agree with observed concentration time-courses over a time frame of ${48}\,\hbox {h}$.

### Fluorescent reporters of Nrf2 signaling components to enable quantitative ODE modeling

**Figure 2:**
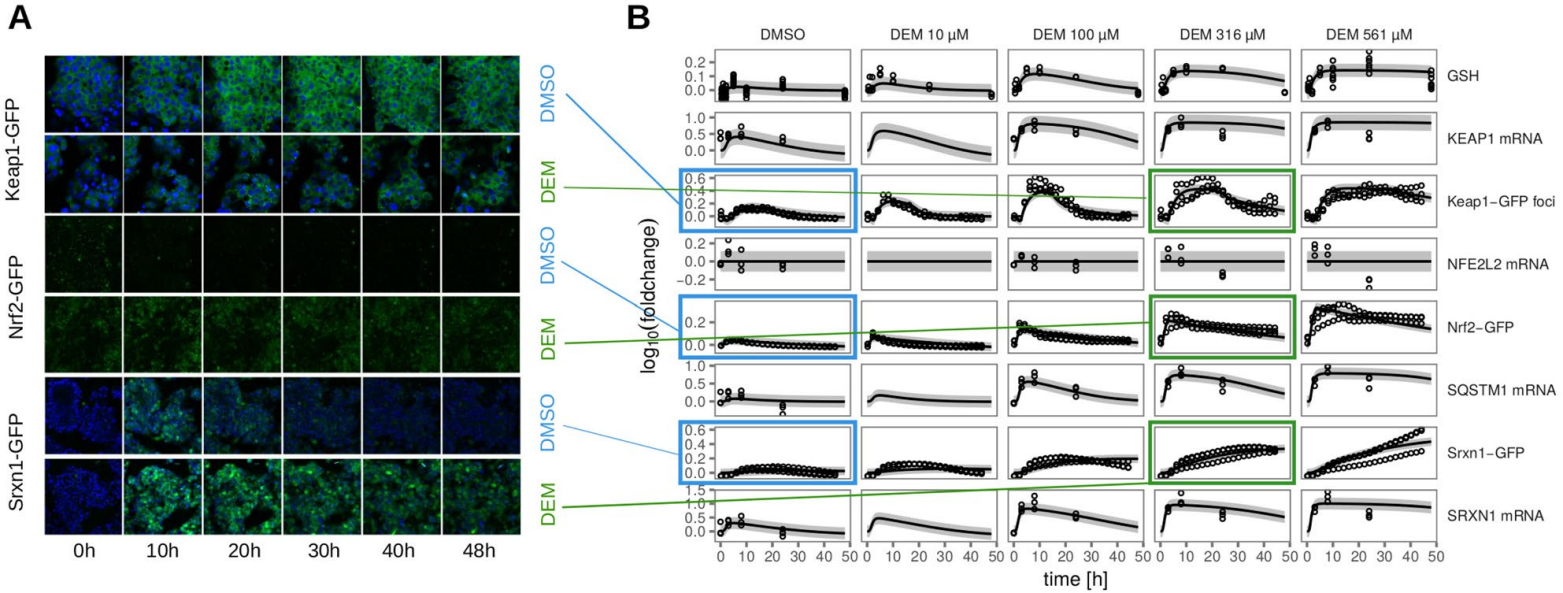
Dynamics of the response to treatment with DEM for 48 h in HepG2 Keap1-GFP, Nrf2-GFP and Srxn1-GFP cell lines. (**A**) Example images of Keap1-GFP, Nrf2-GFP and Srxn1-GFP of 0, 10, 20, 30, 40 and 48 h timepoints for control (DMSO) and ${316}\,\upmu \hbox {M}$ DEM and for DMSO. The Hoechst stained nuclei are blue and the GFP signal is green. They are connected to the corresponding panel of B) by blue (DMSO) or green (${316}\,\upmu \hbox {M}$ DEM) lines. (**B**) 48 h time course exposure with either DMSO or 10, 100, 316 and ${561}\,\upmu \hbox {M}$ DEM for different measurements related to the Nrf2 pathway (total GSH measurements, KEAP1, SQSTM1, NFE2L2 and SRXN1 mRNA, Keap1-GFP foci, Nrf2-GFP and Srxn1-GFP). The circles represent the experimental data and the lines represent the trajectories of the fitted model. Shaded areas around the model prediction indicate one standard deviation as estimated by the error model.

In earlier work, we developed a panel of reporter cell lines of stress response pathway activation, including proteins within the Nrf2 signaling pathway^33^. We exposed cell lines that had either Keap1, Nrf2 or Srxn1 tagged with green fluorescent protein (GFP) to a concentration range of DEM and monitored the response of these Nrf2 pathway components using high throughput confocal imaging (Fig. 2). DEM exposure resulted in a clear accumulation of Keap1-GFP in cytoplasmic autophagosomal foci, an accumulation of Nrf2-GFP in the nucleus and an accumulation of Srxn1-GFP in the cytoplasm, which is in agreement with the current qualitative understanding of the Nrf2 pathway. In addition we measured mRNA levels of the key players in the Nrf2 response: KEAP1, NFE2L2, SQSTM1 and SRXN1 after 3, 8 and ${24}\,\hbox {h}$ of DEM exposure. We observe an upregulation for KEAP1, SQSTM1 and SRXN1 with a peak around ${8}\,\hbox {h}$ after DEM (Fig. 2B; visible for all concentrations). NFE2L2 mRNA levels stay constant after exposure to DEM, indicating that Nrf2 is not positively regulating itself by expression of NFE2L2 mRNA. To assess GSH depletion and production, we also measured the total glutathione levels (GSH and GSSG combined) after 0.5, 1, 2, 5, 10, 24 and ${48}\,\hbox {h}$ of DEM exposure. DEM upregulated total glutathione levels (Figure 2B), which was expected, since expression of GSH producing enzymes such as GCLM is promoted by nuclear Nrf2. To test the literature-based model, we systematically adjusted the various reaction rates by numerical optimization of the likelihood function to minimize the discrepancy between the time-course of the observed concentrations in the fluorescent reporter cell lines and the corresponding model predictions. The result of the parameter estimation is shown in Fig. 2B.

The model fit illustrates that Nrf2 accumulation in the nucleus is early and fast. Specifically, the Nrf2 dynamics are governed by a fast increase during the first 3–5 h followed by a slow relaxation phase over more than 48 h. The Nrf2 mRNA data exhibit no significant increase at high concentrations of DEM. Accordingly, the data is described by a flat model line. In contrast, KEAP1 and SRXN1 mRNA are upregulated within the first 3–10 h. According to the model, upregulation saturates around ${500}\,\upmu \hbox {M}$ of DEM. Similarly, GSH expression is upregulated upon exposure to DEM, rising by up to 30% with increasing DEM concentrations. According to the model fit, GSH amounts are not significantly reduced by its degradation with DEM. Keap1 concentrations in the autophagosome exhibit a small but visible degradation by 5-10% during the first three hours. The model suggests that this decrease is caused by the sequestration of Keap1 in the complex with Nrf2 due to its interaction with DEM. Following translocation of newly formed Nrf2 to the nucleus, KEAP1 mRNA levels, total Keap1 protein concentrations and Keap1 concentrations in the autophagosome increase. Last but not least, Srxn1 protein concentrations exhibit a similar yet more gradual increase as Keap1 protein concentrations. Production of Srxn1 begins as early as three hours upon DEM treatment, but does not reach a plateau within 48 h at DEM concentration of ${561}\,\upmu \hbox {M}$. As a side note, the observed modest response for the DMSO control was also incorporated into the model to take into account that a minor part of the pathway activation observed under the experimental conditions used is due to DMSO rather than DEM (see Supplement).

### Knock-downs deliver detailed insight in Nrf2 signaling

**Figure 3:**
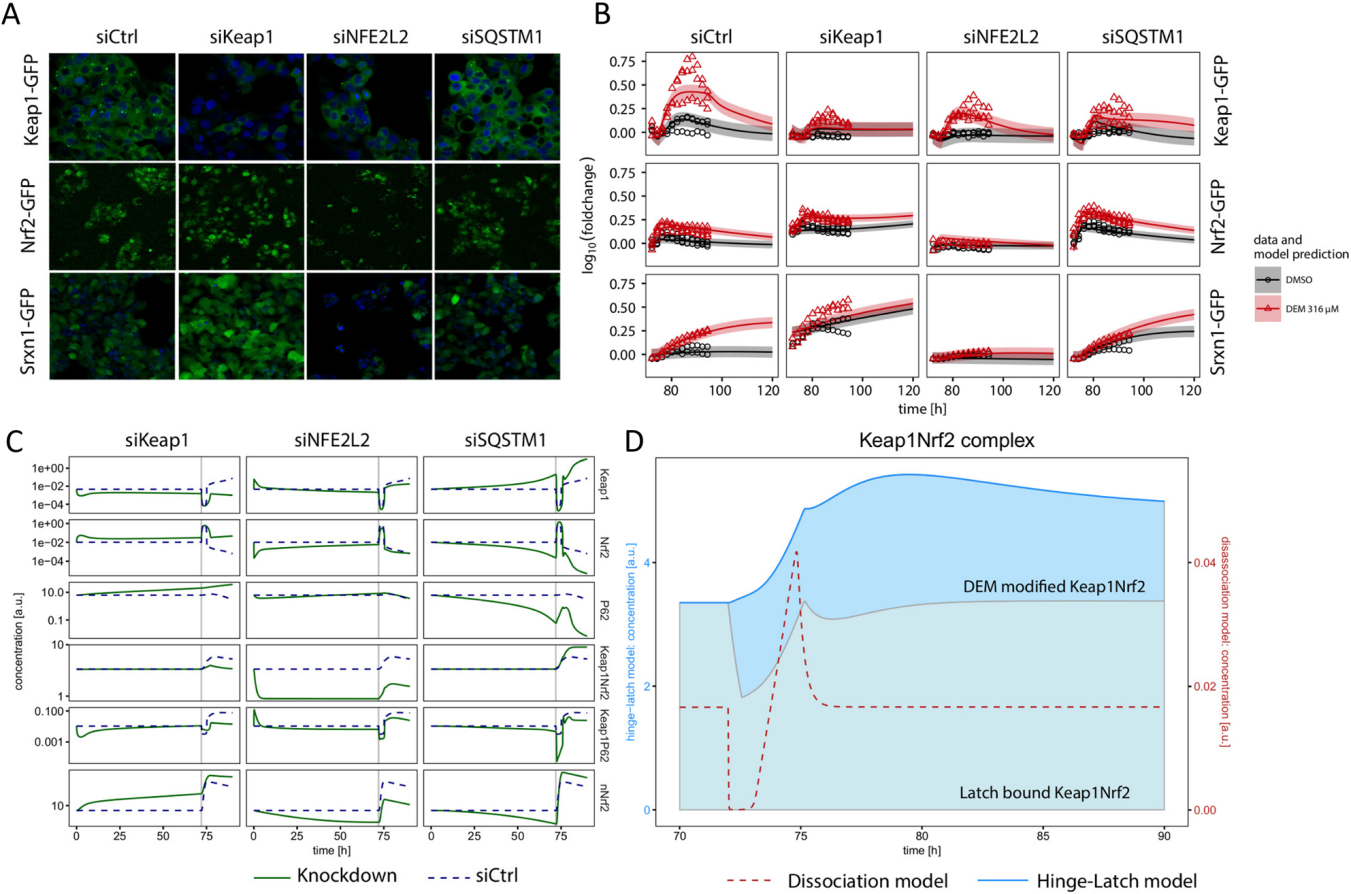
Dynamics after knock down of Keap1, NFE2L2 and SQSTM1 in the Srxn1-GFP, Keap1-GFP and Nrf2-GFP reporter cell lines. (**A**) Example images of Keap1-GFP, Nrf2-GFP and Srxn1-GFP for siCtrl, siKEAP1, siNFE2L2 and siSQSTM1 at the 24 h timepoint. The Hoechst stained nuclei are blue, the GFP signal is green. (**B**) Model prediction (lines) and data points (circles and triangles) for knock-down of siCtrl, siKEAP1, siNFE2L2 and siSQSTM1 in Keap1-GFP, Nrf2-GFP and Srxn1-GFP for DMSO control (black) and DEM ${316}\,\upmu \hbox {M}$ treatment (red). Shaded areas indicate one standard deviation as estimated by the error model. The 72 h of knock-down before drug exposure are not shown. (**C**) Model prediction of the internal states Keap1, Nrf2, p62, Keap1p62, Keap1Nrf2 and nNrf2 for the hinge-latch (HL) model after knock down with siCtrl (dashed lines), siNFE2L2, siKEAP1 and siSQSTM1 (solid lines), including the first 72 h of knock-down simulation. At 72 h DEM ${316}\,\upmu \hbox {M}$ was added (indicated with the vertical line). (**D**) Model prediction for the Keap1-Nrf2 complex for the hinge-latch (HL) model (solid line) and disassociation model (dashed line). The filled areas correspond to the two major model states in the HL-model contributing to the Keap1-Nrf2 complex, i.e., the latch bound Keap1-Nrf2 (Keap1 is bound to Nrf2 at both sites) and DEM modified Keap1-Nrf2 (Keap1 is bound to Nrf2 at only one site due to the modification of Keap1). Contributions to the Keap1-Nrf2 complex from other model states were found to be negligible. At 72 h DEM ${316}\,\upmu \hbox {M}$ was added.

To study the role of the individual players of the Nrf2 pathway in more detail and to assess the role of p62 in Keap1 degradation in our system, we performed knock-downs of KEAP1, NFE2L2 and SQSTM1 by siRNA and evaluated the response of Keap1-GFP, Nrf2-GFP and Srxn1-GFP to ${316}\,\upmu \hbox {M}$ of DEM during ${24}\,\hbox {h}$ treatments (Fig 3A,B). Knock-down of states within the cell can be directly incorporated into the dynamic modeling framework by adjusting the protein production rates of Nrf2, Keap1 and p62 for the corresponding knock-downs. Since siRNA knock-down is never 100% effective, we estimated the modified protein production rates by the model. The model predicts that initial knock-down of Keap1 by siKEAP1 is counteracted by increasing Nrf2 concentrations, which subsequently upregulate Keap1 and Srxn1 mRNA production (Fig 3C). As a consequence, base levels of Keap1-GFP after ${72}\,\hbox {h}$ of knock-down are predicted to be similar to those before, albeit slightly lower. Upon DEM treatment, we observe increased Nrf2-GFP levels, however Keap1-GFP is hardly induced due to the siKEAP1 treatment (Fig 3A,B). Knock-down of Nrf2 by siNFE2L2 slightly decreases Nrf2-GFP, Keap1-GFP and Srxn1-GFP base levels. As expected, treatment with DEM, started at 72 hours after knock-down initiation, leads to a significantly reduced response compared to siCtrl (Fig 3A). The decrease in Nrf2 first leads to an increase of unbound Keap1, due to the reduced complex formation with Nrf2 (Fig 3C; middle panels). Subsequently, this effect is reversed by the decreasing Nrf2 levels in the nucleus (nNrf2), leading to a decreased Keap1 level that is accompanied by a recovery of unbound Nrf2 because of a decreased sequestration in complexes with Keap1 (Fig 3C; middle panels). Finally, the model predicts that p62 knock-down by siSQSTM1 yields lower base levels of Keap1-GFP in the autophagosome and also lower levels upon DEM treatment relative to the siCtrl condition (Fig 3C; right panels). This observation is consistent with the role of p62 in the degradation of Keap1 in autophagosomal foci. As a consequence of the low p62 base levels, Keap1 base levels increase and nuclear Nrf2 base levels decrease (Fig 3C). In conclusion, the experimental data are well described by the model for all knock-downs, except for slight deviations for siKEAP1, where the increase in Keap1-GFP and Srxn1-GFP after DEM exposure was underestimated by the model.

### Internal states of the Nrf2-Keap1 complex support the hinge-latch hypothesis

In the current literature the hinge-latch hypothesis is the leading hypothesis with much evidence pointing in that direction^2,3,23,34^. However, there is also data supporting the so-called dissociation hypothesis^3,35^, in which Nrf2 is released from Keap1 after introduction of oxidative stress. The released Nrf2 is then able to translocate to the nucleus alongside the newly synthesized Nrf2. In the hinge-latch hypothesis, the conformation of the Nrf2–Keap1 complex is altered upon oxidative stress, implying that induction of target genes is only facilitated by newly synthesized Nrf2. To test whether our model is able to distinguish between the two competing hypotheses, we adapted the model to represent the dissociation hypothesis (Supplement Section 2). This model was also able to describe the experimental data (Supplement Fig. S5). Therefore, based on the current data, we are not able to exclude either hypothesis. Nevertheless, if we compare the internal states corresponding to Nrf2–Keap1 complexes (Fig 3D), the dissociation model predicts an immediate decrease in the complex upon exposure to DEM followed by a sharp increase in complex formation, while the hinge-latch model predicts a prolonged increase in the complex that starts immediately upon oxidative stress. This observation in the model predictions indicates that our model supports the hinge-latch hypothesis^36^. Specifically, this hypothesis was supported by measurements of the Nrf2–Keap1 complex, demonstrating that the complex remains present rather than diminished upon oxidative stress. This is also the behavior we obtain with the hinge-latch model, but not with the dissociation model.

### Diclofenac and omeprazole based validation of the Nrf2 model

**Figure 4:**
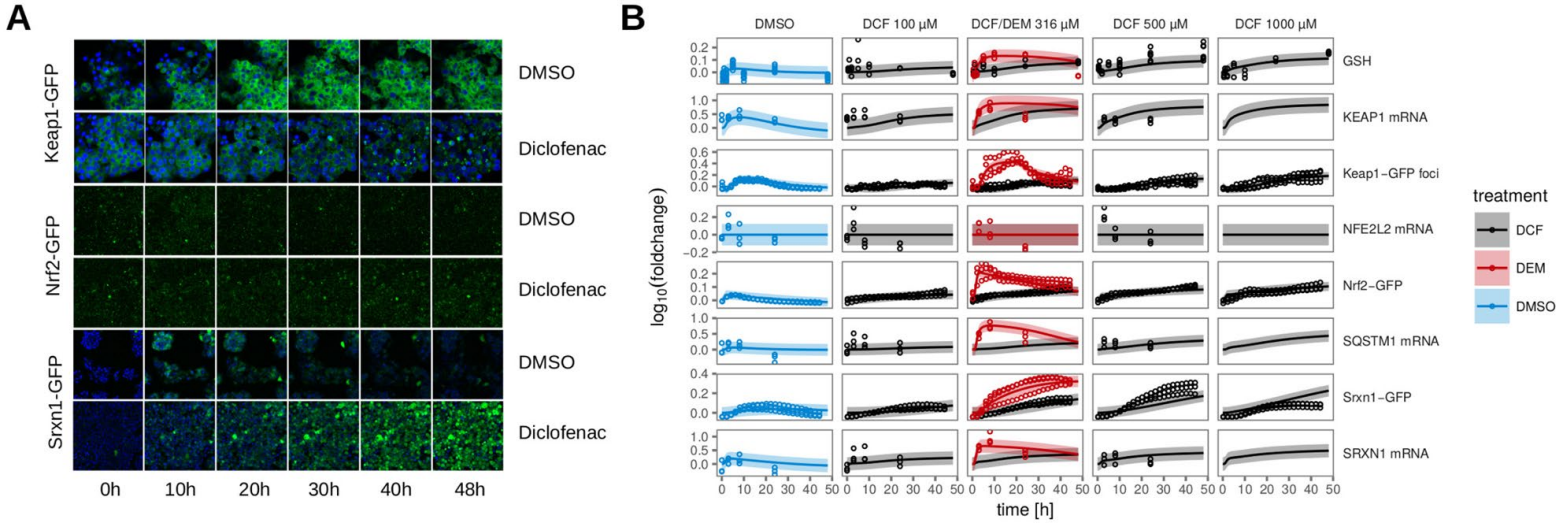
Response dynamics to 48 hours of treatment with DCF in Keap1-GFP, Nrf2-GFP and Srxn1-GFP cell lines. (**A**) Example images of Keap1-GFP, Nrf2-GFP and Srxn1-GFP of 0, 10, 20, 30, 40 and 48 hours timepoints for ${500}\,\upmu \hbox {M}$ DCF or DMSO. The Hoechst stained nuclei are blue, the GFP signal is green. (**B**) 48 h time course upon exposure with just DMSO or with 100, 316, 500 and ${1000}\,\upmu \hbox {M}$ DCF for different measurements of the Nrf2 pathway (total GSH measurements, KEAP1, SQSTM1, NFE2L2 and SRXN1 mRNA, Keap1-GFP foci, Nrf2-GFP and Srxn1-GFP). For comparison, also the DMSO control and ${316}\,\upmu \hbox {M}$ DEM results are shown in red and blue, respectively. The dots represent the experimental data and the lines represent the model based estimates. Shaded areas around the model prediction indicate one standard deviation as estimated by the error model.

Since we are interested in the role of Nrf2 signaling in DILI, we set out to validate the constructed model as based on the general ROS-inducer DEM with two DILI inducing compounds. Specifically, we used DCF, a drug often prescribed as a non-steroid anti-inflammatory drug, and OMZ, a proton pump inhibitor used for gastric acid inhibition^37^, both known to induce idiosyncratic DILI. Previously, we showed that DCF induces several Nrf2 target genes^9^. Therefore, we exposed Keap1-GFP, Nrf2-GFP and Srxn1-GFP expressing HepG2 cells to 100, 316, 500 and ${1000}\,\upmu \hbox {M}$ of DCF. In addition, we measured mRNA levels of SQSTM1, NFE2L2, KEAP1 and SRXN1 and GSH similar to the DEM case. Next, we refitted the parameters of the hinge-latch based ODE model to all experimental data including the data following DCF exposure. A few parameters were added in order to describe the DCF specific association and degradation rate constants, while all other parameters were considered not to differ between DEM or DCF treatment. The model can generally describe also the DCF data (Fig 4), apart from small deviations for the mRNA data (for more information, see Supplement Section 1.3). To test whether the model could also describe data for the other investigated DILI compound OMZ, we exposed Nrf2-GFP and Srxn1-GFP expressing HepG2 cells to 4.7, 23.5, 47, 94, 188 and ${282}\,\upmu \hbox {M}$ of OMZ. As for DCF, some parameters were added in order to describe OMZ specific association and degradation rate constants, while all other parameters were considered not to differ between DEM, DCF or OMZ treatment. This gave a reasonable fit for the OMZ data. Interestingly, when we allowed also the degradation rate of Srxn1-GFP and the Nrf2-dependent production rate of SRXN1 mRNA to be refitted, there was a significant improvement (for more information, see Supplement Section 1.3), suggesting that the regulation of Srxn1 by Nrf2 may be different for OMZ than for DEM and DCF. In summary, our analysis confirms that our quantitative ODE model of the Nrf2 signaling pathway is able to explain cellular perturbations following a general ROS inducer and drugs known to induce severe liver injury.

## Discussion

DILI remains a major problem both in the clinic and in drug development. Adaptive stress response proteins are upregulated after cellular stress and can therefore be indicative for DILI development. Nrf2 is one of the prominent adaptive stress responses, since in many circumstances reactive oxygen species are being formed. Therefore, Nrf2 is key in the cellular defense after drug exposure. Dynamic quantitative ODE modeling can help to predict the unobserved processes of the Nrf2 signaling pathway and its reaction to oxidative stress inducers. Therefore, we set out to construct an ODE model of the Nrf2 signaling pathway describing the reactions of the individual players within the pathway. Nrf2 can be activated in several different ways: via cysteine residue binding of Keap1, via phosphorylation of the Nrf2 protein or via interaction with different binding partners or miRNAs^38–42^. To be able to deal with different types of activation we chose to work with a general model of Nrf2 activation. We were able to build a model based on exposure to DEM and confirm the model with knock-down of different components and exposure to the DILI drugs DCF and OMZ.

In most cases, the fits of the model showed proper resemblance with the experimental data. However, knock-down of KEAP1 did not result in the predicted levels of Srxn1-GFP. The model predicted a small effect of DEM due to the decrease of Keap1, yet in the experiment DEM resulted in higher levels of Srxn1-GFP. One explanation for this might be that other factors inhibit Srxn1 expression in absence of Keap1. Thus, these findings warrant further research, which could lead to additional mechanistic understanding of the Nrf2 pathway. Our analysis incorporating either the hinge-latch or the dissociation hypothesis into our models showed that both hypotheses could fit the currently presented data. However, studying the internal states of the Nrf2–Keap1 complex in both models point in the direction of the hinge-latch hypothesis. In 2006, Kobayashi et al. demonstrated that the Nrf2-Keap1 complex was indeed present after introduction with the oxidative stress inducer tBHQ for 12 h using immunoprecipitation^34^. This observation led to the introduction of the hinge-latch hypothesis and here we showed that this is indeed consistent with prolonged presence of the Nrf2-Keap1 complex.

The relative increase in Nrf2 protein levels we measured in our HepG2-GFP cells with imaging upon exposure to compounds such as DEM has previously been observed in absolute amounts in various murine cell types^43^. However, in that study no significant change in Keap1 protein levels occurred, whereas we did find such an increase at both mRNA and protein level. Because our Keap1 measurement at protein level was based on the mean number of Keap1 foci per cell, we cannot fully exclude that the overall Keap1 level within cells remained constant, and that there was only a focal Keap1 increase due to local accumulations. Nevertheless, given our finding of a KEAP1 increase also at mRNA level, we consider it likely that the total amount of Keap1 protein did indeed increase in our studied setting. Because in the study by Iso et al.^43^ the Keap1 protein level was measured at 3 hours post exposure, and because we observed a delay of a few hours for the increase of the number of Keap1 foci, the 3-h time point may have been too early for increased Keap1 protein levels to manifest. As another alternative explanation for the difference in observations, the Keap1 increase may be HepG2 specific.

In the future our current model can be used to predict the response of additional Nrf2 signaling components. In addition, this model can be extended with components directly involved in cell death. Our current model only describes the adaptive part of the Nrf2 pathway, but sustained activation could result in more severe damage to mitochondria potentially leading to cell death and at the organ level the onset of e.g. liver failure. Furthermore, the model can be used as a tool to study potential mechanisms involved in the response to repeated exposures of compounds or to study relationships between the structural similarity of compounds and mechanism of Nrf2 activation. In this way, this model can both contribute to a better understanding of the Nrf2 signaling pathway and of DILI.

## Materials and methods

### Reagents

DEM, DCF and OMZ were obtained from Sigma (Zwijndrecht, The Netherlands) and were dissolved in DMSO. Hoechst 33342 (Thermo Scientific, Leiden, The Netherlands) 200 ng/ml was used to stain nuclei of live cells.

### Cell line

Human hepatoma cell line HepG2 was obtained from the American type tissue culture collection (ATCC, Wesel, Germany). HepG2 cells were cultured in phenol red Dublecco’s modified Eagles medium (DMEM) supplemented with 10 (v/v) fetal bovine serum (FBS), 25 U/ml penicillin and 25 $\upmu$g streptomycin (PSA, Invitrogen). Note that all experiments performed with the HepG2 cells (imaging, knockdowns, qPCR, GSH measurements) were executed with at least three biological replicates.

### GFP BAC reporter generation

Human SRXN1, NFE2L2 (Nrf2), KEAP1 were selected and tagged with enhanced Green Fluorescent Protein (GFP) as described previously using the bacterial artificial chromosome recombineering technique (BAC)^44^ and stably introduced into HepG2 cells by transfection and 500 $\upmu$g/ml G418 selection as reported previously^45^.

### siRNA transfections

Transient knock-downs were achieved using siGENOME siRNA reagents of siSQSTM1, siKEAP1, siNFE2L2 and siCONTROL1 (50 nM; Dharmacon ThermoFisherScientific, Landsmeer, The Netherlands). HepG2 cells were transfected with INTERFERin (Polyplus transfection, Leusden, The Netherlands). Cells were incubated for 72 hours with siRNA before compound treatment and microscopy.

### Microscopy

The GFP intensity levels of reporter cell lines were measured using a Nikon TiE2000 confocal laser microscope equipped with an automated stage, perfect focus system and live cell control to ensure 37$^\circ$C and CO$_2$ conditions during imaging. Images were acquired with 20X objective (NA = 0.75, Violet Corrected). Images were obtained every hour during 48 hours, using 405 and 488 lasers detecting Hoechst 33342 and GFP respectively.

### GSH measurements

Cells were seeded at a density of 200.000 cells/well in a 24-wells format and incubated for 48 h under a 37 $^{\circ }$C and humidified 5% CO$_2$ atmosphere environment. After 48 h of incubation the cells were exposed to either DEM or DCF for 0.5, 1, 2, 5, 10, 24 or 48 h. After exposure the cells were lysed using 10 mM HCl and scraped cells were transferred to eppendorf tubes. Proteins were diluted and precipitated by using 1.3% 5-sulfosalicyclic acid. Dilutions were incubated for 5 min on ice and subsequently centrifuged at 4000 rpm at 4 $^{\circ }$C for 5 min. GSH stock buffer (consisting of EDTA (ehylenediaminetetraacetic acid, Sigma-Aldrich, Zwijndrecht, Netherlands), monosodium phosphate (Sigma-Aldrich, Zwijndrecht, Netherlands) and MilliQ) was added to supernatant to receive measurement sample. The supernatant was transferred to a 96-well plate and supplemented with Daily Assay Reagent (DNTB (5,5’-dithios-(2-nitrobenzoic acid)) and NADPH (nicotinamide adenine dinucleotide phosphate) in GSH stock buffer). After 5 min of incubation at room temperature GSH Reductase (Sigma-Aldrich, Zwijndrecht, Netherlands) was added to the sample. The absorption was measured at 405nm using the Fluostar Optima (BMG LabTech, Ortenberg, Germany). To measure the protein concentration cell lysate was added to MilliQ. After Bio-Rad Reagens (Bio-Rad Laboratories GmbH, Munchen, Germany) was added. The plate was then incubated for 5 min at room temperature before measuring the absorption at 590 nm using the Fluostar Optima (BMG LabTech, Ortenberg, Germany).

### qPCR analysis

Cells were seeded with a density of 200.000 cells/well in a 24-wells format and incubated for 48 h under a 37 $^{\circ }$C and humidified 5% CO$_2$ atmosphere environment. After 48h of incubation the cells were exposed to either DMSO, diethylmaleate or diclofenac for 3, 8 or 24h. After exposure cells were lysed and RNA was isolated using the RNA isolation kit (Macherey Nagel GmbH, Düren, Germany). RNA concentrations were measured by nanodrop and cDNA was constructed using the REVERTAID H MINUS cDNA kit (Thermo Scientific, Leiden, The Netherlands). Kicqstart primers for TBP, KEAP1, NFE2L2, SRXN1 and SQSTM1 (Sigma Aldrich, Zwijndrecht, The Netherlands) were used to detect mRNA levels using POWRUP SYBR master mix (Thermo Scientific, Leiden, The Netherlands). Cycle time values were obtained from a QuandStudio 6 Flex qPCR machine (Thermo Scientific, Leiden, The Netherlands). Cycle time values exceeding the threshold were normalized to the housekeeping gene TBP and used as a quantification of the qPCR.

### Reporter response quantification

Reporter response quantification was conducted similar as in Wink et al.^45^. Mean nuclear GFP intensity was used as a quantification for Nrf2-GFP, integrated cytoplasmic GFP intensity was used as a quantification for Srxn1-GFP and amount of cytoplasmic foci per cell was used as a quantification for Keap1-GFP.

### Data preprocessing

Time-course data from independent, biological replicates were aligned to yield average time courses. We use a scaling model to account for systematic differences between independent experiments, due to e.g., the number of cells per well or the average light intensity. The signal $S_{i, k, n}$ for the time point $t_n$, the experiment *k* and the experimental condition *i* (treatment or knock-down) is described by the model $S_{i, k, n} = \frac{y_{i, n}}{s_k}$, where $y_{i, n}$ refers to the time-course parameters and $s_k$ indicates the scaling factors. We assume that noise of the measured signals is log-normally distributed with constant standard deviation. The time-course parameters $y_{i, n}$ represent the average dynamics over all biological replicates. In this way, the experimental data is condensed and replicates are replaced by single time courses for dynamic modeling.

This procedure has been applied to the following two independent series of experiments: (1) time-course experiments for the concentrations 0, 10, 100, 316 and ${561}\,\upmu \hbox {M}$ of DEM, for 100, 316, 500 and ${1000}\,\upmu \hbox {M}$ of DCF, and for 4.7, 23.5, 47, 94, 188 and ${282}\,\upmu \hbox {M}$ of OMZ, respectively, and (2), time-course experiments for knock-down of Keap1, Nrf2 and p62 expression by siRNA and treatment with either 0 or ${316}\,\upmu \hbox {M}$ of DEM.

Finally, to align the two series of experiments, a mixed scaling-offset model has been applied. The model equations read $S_{i, k, n} = \frac{y_{i, n}}{s_k} + b_k$, where $b_k$ refers to a systematic offset between the two series. The result of the alignment procedure is a set of time-resolved data for all experimental conditions that share a common scale and are therefore comparable. The time-course parameters after the two alignment steps are employed for subsequent dynamic modeling.

### Dynamic modeling

The Nrf2 signaling pathway is described by a chemical reaction network that is mostly based on mass-action kinetics. Some reactions, like the production of mRNA, are modeled by Hill kinetics. The reaction network is translated into a set of ordinary differential equations (ODEs) that need to be solved numerically to describe the time-evolution of the species concentrations. The ODEs depend on parameters like rate constants, Hill-coefficients and initial concentrations of species.

The system is assumed to be in equilibrium at $t = 0$. This steady-state assumption induces relations between the rate constants and initial concentrations that, in principle, allow to compute initial concentrations from rate constants. The resulting equations for the Nrf2 signaling network are polynomials of higher order which cannot be solved analytically. We use the approach presented in^46^ to avoid the problem of higher-order polynomials and use the steady-state constraint to reduce the number of free parameters.

Parameters associated with siRNA knock-down, i.e., the production rates of proteins Keap1, Nrf2 and p62 are implemented as a switch that is triggered by the knock-down associated with the corresponding experiment. Since the system is in a steady-state at $t = 0$, the knock-down switch induces a time-dependent shift of all concentrations. The knock-down is simulated over ${72}\,\hbox {h}$. After ${72}\,\hbox {h}$, the cells are treated with DEM or DCF. The treatment is modeled by a Gauss-shaped bolus which is switched on after $t = {72}\,\hbox {h}$. The height of the Gauss curve corresponds to the DEM/DCF concentration.

To compare simulated concentration time-courses with measured time-course data, we include the unknown concentration scale of fluorescence microscopy measurements and possible background fluorescence into an observation function. The scaling factors and offsets constitute additional parameters that need to be determined from data. In addition, we specify the expected measurement noise assuming a log-normal distribution with constant standard deviation for each measured target. In summary, the structure of the observation is given by

1 $$\begin{aligned} y = \log (s\cdot x + b) + \epsilon , \end{aligned}$$

where *y* denotes the logarithm of the data, *x* denotes the model prediction, *s* and *b* reflect scaling and offset parameters and the noise $\epsilon \sim N(0, \sigma ^2)$ is a normally distributed random variable with standard deviation $\sigma$.

The free parameters are determined from the experimental data. For parameter estimation, the maximum-likelihood approach is used. The joint likelihood reads

2 $$\begin{aligned} -2\log L(\theta )&= \sum _i \left( \frac{y(t_i,\theta ) - y_i^D}{\sigma _i(\theta )}\right) ^2 + \log (\sigma _i(\theta )^2), \end{aligned}$$

where $y(t_i, \theta )$ is the observation predicted by Eq. (1) at time point $t_i$ given the parameters $\theta$. The symbol $y_i^D$ denotes the data point at time point $t_i$ and $\sigma _i(\theta )$ is the standard deviation computed from the error model, in our case a constant for each target. Parameter estimates are obtained by minimizing Eq. (2) with respect to $\theta$.

Frequently, parameters are found to be non-identifiable. One source of non-identifiability is symmetries. We use a Lie-group approach^47^ to determine all possible scaling- and translation symmetries and reduce the number of free parameters accordingly. Another source of non-identifiability is lack of informative data. We use the profile-likelihood approach as presented in^48^ to determine non-identifiable parameters and compute confidence intervals. Methods presented in^49^ are used to further reduce the model and obtain identifiable parameters.

For a detailed summary of all model equations, steady-state constraints, observation functions and model parameterization see Supplement. The model is available in PEtab format^50^ at https://github.com/JetiLab/Nrf2-signaling.

All model analyses, parameter estimation and identifiability analysis have been carried out using the dMod^51^ package for R. The package is available on the Comprehensive R Archive Network (CRAN).

## Supplementary Information

Supplementary Information.

## Data Availability

The datasets generated during and/or analysed during the current study are available from the corresponding author on reasonable request. The model is available at https://github.com/JetiLab/Nrf2-signaling.
