# Supplementary material for: Dynamic modeling of Nrf2 pathway activation in liver cells after toxicant exposure
Source: Sci Rep. 2022 May 5;12:7336. doi: 10.1038/s41598-022-10857-x (PMC9072554; doi:10.1038/s41598-022-10857-x)
Supplement: Supplementary file 1 — Supplementary Information. [file 41598_2022_10857_MOESM1_ESM.pdf]

Supplement: Dynamic modeling of Nrf2 pathway activation in liver cells after toxicant exposure

Steven Hiemstra[1,+], Mirjam Fehling-Kaschek[2,+], Isoude Kuijper[1,+], Luc Bischoff[1], Jeroen Esselink[1], Allard van Egmond[1], Jornt Mos[1], Joost Beltman[1], Jens Timmer[2,3,*], Bob van de Water[1,‡], and Daniel Kaschek[2,‡]

[1]Division of Drug Discovery and Safety, Leiden Academic Centre for Drug Research, Leiden University, Leiden, The Netherlands
[2]University of Freiburg, Institute of Physics, Freiburg, Germany
[3]University of Freiburg, BIOSS Centre for Biological Signalling Studies, Freiburg, Germany
[*]jeti@fdm.uni-freiburg.de
[+]these authors contributed equally to this work
[‡]these authors also contributed equally to this work

ABSTRACT

Further information is provided for both models. This includes a full list of ODEs, obervation functions and parameter transformations. The mode reduction is discussed for the model based on the hinge-latch mechanism.

1 Hinge Latch Model

1.1 Model Description

All reactions implemented in the HL model are provided in Table 1. The key part of the model describes the interactions of Keap1, Nrf2 and P62. The interactions in absence of drugs are given in reactions 1-7: The HL-mechanism is implemented by a two-step binding of Nrf2 to the Keap1 homo-dimer, denoted by Keap1, first attaching Nrf2 to one Keap1 unit building the Keap1Nrf2 complex (reaction 1) followed by a step attaching the end of Nrf2 to the second Keap1 unit transforming the complex to Keap1Nrf2L (reaction 2). The degradation of Nrf2 is triggered only for the fully attached Keap1Nrf2L complex and leads to a restitution of free Keap1 (reaction 3). P62 can bind to a Keap1 unit building the Keap1P62 complex (reaction 4) which is transported to the autophagosome (reaction 6) and degraded (reaction 7). Since the Keap1Nrf2 complex still offers one free Keap1 unit, P62 can also attach to Keap1Nrf2. In the model we do not distinguish between the Keap1P62 complex with or without Nrf2 attached and we consider both complexes to be transported to the autophagosome and to be degraded irrespective of Nrf2 (yielding reaction 5).

The production and degradation processes for the proteins of interest are given in reactions 8-30. A basal production of cytoplasmic Nrf2 is assumed, which is decreased upon Nrf2 siRNA knockdown (reaction 8). This change of the production rate is implemented by the switch variable Ko_Nrf2 which is set to one for the knockdown condition, resulting in the rate buildNrf2Base_Ko, and to zero otherwise, resulting in the rate buildNrf2Base. The import and export of Nrf2 to and from the nucleus (reactions 9-10) play an important role for the production of the other proteins that are triggered by nuclear Nrf2, nNrf2. The production of Keap1 (reactions 11-15), P62 (reactions 16-20), Srxn1 (reactions 20-25) and GSH via GCL (reactions 26-30) are each described by the same type of reactions:

- mRNA production with a basal rate
- mRNA production induced by nNrf2, implemented by Hill-kinetics
- mRNA degradation
- protein production proportional to the mRNA level
- protein degradation

Switches for the Keap1 and P62 siRNA knockdown are implemented in analogy to the Nrf2 siRNA knockdown. The production of monomeric Keap1 and its dimerization are modeled by a single reaction. Furthermore, it should be noted, that Srxn1 does not feed back into the signaling cascade but serves as a read-out of nNrf2. The role of GSH is discussed in the following paragraph

on drug-induced reactions.
In the experimental setup, the cells were treated with either DEM, DCF or OMZ. For the siRNA knock-down experiments, the knock-down is simulated for 72 hours before treatment with the compound. Accordingly, the knock-down control conditions are shifted by 72 hours to match the time frame of the knock-down conditions. The additional reactions due to the drug treatment are given in reactions 31-42. The input of reactive metabolites RM (DEM, DCF or OMZ) is implemented as a Gaussian peak around the input time with a height proportional to the dose (reaction 31). To avoid drug effects in the simulation before the actual treatment time, the Gaussian is multiplied by the switch variable DRUG, changing from 0 to 1 at $t = 72$ h. Since the data suggests that a drug effect is also induced in the absence of RM, it was concluded that this might be due to Reactive Oxygen Species (ROS) induced by cellular stress due to DMSO. In analogy to RM, an independent input for ROS was implemented (reaction 32). Its strength (dose_ROS) was coupled to the amount of DMSO for each condition, since the drugs were dissolved in DMSO and then dilluted to get different drug concentrations. While RM is only degraded via GSH (reaction 33), the degradation of ROS is implemented as a purely time-dependent effect (reaction 34). The mode of action of the drugs is the change of conformation of Keap1 to Keap1Mod (reactions 35-38). The idea of the HL-mechanism is that the modified dimer is unable to attach both ends of Nrf2. Therefore, it was implemented such that the second binding step (reaction 2) is prevented, while the first binding might still occur. In the model, RM and ROS not only affect the free Keap1 dimers (reaction 35-36) but also modify Keap1Nrf2 (reaction 37-38). Nrf2 can still bind to the first unit of the modified Keap1 dimer (reaction 40). It is assumed that the modification does neither alter the Keap1 degradation (reaction 39) nor the P62 binding (reaction 41-42), which are implemented with the same rate constants as for the unmodified Keap1.
Summarized, the model comprises 18 states and 66 parameters, including initial value parameters for the states. The resulting ODEs for the states are provided in Table 2.

1.1.1 Observation Function

The states described by the model are connected to the measured data by an observation function, given in Table 3. A scaling parameter, denoted by s_*state*, is used to account for the unknown normalisation of the fluorescense microscopy and qPCR data. In addition an offset, denoted by off_*state*, is added for the fluorescence microscopy data to account for the background fluorescence. The mRNA level of Nrf2 is modeled as a constant, denoted by const_NFE2L2. The noise of the log-data is normally distributed with constant but unknown standard deviation for each target. To match the model with the log-data, also the model prediction is logarithmized. The unknown standard deviations constitute the error model. In total, 20 parameters are added to the model due to the observation function and error model.

1.1.2 Steady-State and Symmetry Constraints

The system is assumed to be in equilibrium ($\frac{\mathrm{d}}{\mathrm{d}t}\vec{x}(t=0) = 0$) prior to treatment leading to a reduced number of parameters. The resulting 14 substitutions of parameters are given in Table 4. All state names occuring in the equations represent the corresponding initial value parameter of the state.

A further reduction of model parameters is achieved by evaluating the symmetries of the system. The model admits six independent scaling-symmetries. Without loss of generality, from each symmetry group, one parameter can be fixed to an arbitrary, non-zero value. The following reduction was chosen:

$$\text{s\_Srxn1} = \text{s\_mKeap1} = \text{s\_mP62} = \text{s\_mSrxn1} = \text{s\_Gsh} = \text{buildGsh} = 1.$$

1.1.3 Parameter Transformations

The remaining parameters are estimated from the data via the maximum-likelihood method. Since all parameters have by definition positive values and may vary over orders of magnitude, they are log-transformed. To ensure that the knockdown production rates (see eqs. 8, 14, 19) are at least reduced to half the normal production rates, the following transformations are used

$$\text{buildNrf2Base\_KO} = \text{buildNrf2Base} \cdot 0.5 \cdot \exp\left(-\text{Delta\_buildNrf2Base\_KO}^2\right)$$
$$\text{buildP62\_KO} = \text{buildP62} \cdot 0.5 \cdot \exp\left(-\text{Delta\_buildP62\_KO}^2\right)$$
$$\text{buildKeap1\_KO} = \text{buildKeap1} \cdot 0.5 \cdot \exp\left(-\text{Delta\_buildKeap1\_KO}^2\right)$$

and the Delta-parameters are estimated by the fit. To ensure, that the KO rate is always smaller than the unperturbed rate, the Delta-parameters are squared. Furthermore, the reduction of the rates is considered to be at least 50% factor 0.5 in the equations. Similarly, it is assumed that the Keap1P62 complex formation rate for free Keap1 is greater or equal to the rate for Nrf2 bound Keap1:

$$\text{buildKeap1P62} = \text{buildKeap1P62H} + \exp(\text{Delta\_buildKeap1P62})$$

In addition the import rate of Nrf2 to the nucleus is related to the export rate by the ratio parameter alphaNuc:

$$\text{importNrf2} = \exp(\text{alphaNuc})\exp(\text{exportNrf2}) \cdot 0.0638$$

The factor of 0.0638 gives the ratio of the nuclear and cytoplasmatic volumes[1] such that alphaNuc corresponds to the relative steady state Nrf2 concentrations in the nucleus and cytoplasma.

1.1.4 Additional Constraints

Additional information was gathered regarding the protein and complex concentrations in the cells. They are implemented as constraints in the model, by defining log-ratios r_1, r_2, and a ratio r_3 that are pulled to zero via a quadratic prior that is added to the objective function.

1. little free Keap1 compared to P62
   r1 = log((Keap1 + P62) / P62)

2. most Nrf2 stored in complex
   r2 = log((Nrf2 + Keap1Nrf2) / Keap1Nrf2)

3. equilibrium of free and cytoplasmic Nrf2 in the nucleus
   r3 = 1 / alphaNuc

1.2 Optimization and Model Reduction

The objective function is set up as described in the main text (Methods section). For the model selection and reduction part of the analysis a weak prior $(p-\mu)^2/5^2$ centered around $\mu = -1$ was added for each parameter p in order to facilitate convergence of the optimization. Furthermore, the priors for the ratios defined in 1.1.4, $(r_i - 0)^2/0.1^2$ for $i \in \{1,2,3\}$, are added.
The datasets used in the analysis are summarized in Table 6. They are split in four subsets: DEM timecourse, DEM knockdown, DCF timecourse and OMZ timecourse. The model was first calibrated on the DEM timecourse data standalone. It was found that the model could well describe the observed timecourses. However, many local optima were found that describe the data equally well. Additional information is given by the DEM knockdown data which is included in any results from here on. The DCF and OMZ data serve to validate the model and therefore it is not used for the initial model calibration and reduction.
About 2000 fits with random initial parameters $p_i \sim \mathcal{N}(0,5)$, were conducted for the DEM timecourse and knockdown datasets. Only few fits converged to the best optima. To gain a better understanding on these optima, fits were restarted by distributing the parameters around the parameters of the 30 best optima (about 50 fits were started per optimum, giving a total of about 1500 fits). In Fig. 1 A the ordered objective values for the 400 best converged restarted fits are shown. The corresponding parameter values are shown in Fig. 1 B. As a side note, the optima were obtained with parameter priors added to the objective function, such that non-identifiable parameters are strongly restrained by the prior.

In order to investigate parameter identifiability, parameter profiles were calculated for the 10 best optima, shown in Fig. 2. Non-identifiable parameters were tested regarding their correlations to other parameters and gradually fixed to make the model identifiable. After each reduction step, the model was recalibrated and new parameter profiles were calculated. In summary, the following reductions were implemented:

1. Parameters with no lower bound that did not couple to other parameters for small values were fixed to zero, corresponding to the removal of processes not needed to describe the data:
   degradKeap1 = degradP62 = off_Srxn1 = 0

2. The practically non-identifiable scaling factor for nSrxn1 was fixed to one:
   s_nNrf2 = 1

3. Hill-parameters that were reaching their upper bound for all fits were fixed to the upper bound:
   hill_Keap1 = hill_Gcl = hill_P62 = 5

4. The parameter describing the second binding of Nrf2 to the second Keap1 unit was found to be large with no upper bound, corresponding to a fast binding compared to other processes in the model. The parameter was fixed to a value corresponding to a ms timescale:
   build2Keap1Nrf2 = 60*60*1000

5. The GSH degradation and Keap1 modification parameters were also found to be without upper bound and were fixed to a large value of 1000:
   degradGsh = changeKeap1_RM = 1000

6. Either the importKeapP62 or the degradKeap1P62 2 parameter approached large values without constraint, while the other was small and identifiable. At equilibrium, the ratio of the two parameters gives the ratio of the Keap1P62 amount in the autophagosomes compared to that in the cytoplasm: importKeapP62/degradKeap1P62 = aKeap1P62/Keap1P62. In conclusion, two solutions were found by the model, one with more Keap1P62 complex in the cytoplasm and one with more complex in the autophagosomes. Considering concentrations instead of amounts, a much higher concentration of the complex was experimentally observed in the autophagosomes. Assuming that the volume of the autophagomes V_a is about 0.5% to 5% of the cytoplasmic volume V_c[2], we obtain the condition:

   $$\text{importKeapP62}/\text{degradKeap1P62} = \text{aKeap1P62}/\text{Keap1P62} \gg V_a/V_c \approx (0.005-0.05)$$

   Solutions with importKeapP62 $\ll$ degradKeap1P62 were excluded by fixing importKeap1P62 to 20

7. At this point two solutions were found for the ROS based activation, resulting in two groups of parameter values for the correlated changeKeap1_ROS, dose_ROS and degradROS parameters. Without further knowlegde on this possible DMSO based ROS production the solution resulting in slightly better objective values was chosen, by fixing the changeKeap1_ROS parameter:
   changeKeap1_ROS = 0.001

In a final step, the reduced model was fitted without adding the parameter priors to the objective function. The ordered values of the objective function and the corresponding parameter values are shown for the 200 best converged fits in Fig. 3. The 200 fits had a similar objective and parameter values. However, taking a closer look, four parameters still show significant deviations between the different fits. The largest deviation occurred for the parameters dose_ROS and degradROS. The global optimum, taken as the final result, is reliably found in 70 of 200 fits. Parameter profiles for the global optimum are shown in Fig. 2 and the parameter values are given in Table 5.

1.3 Application of model to diclofenac and omeprazole

Our initial expectation was that when we would test the developed model on DCF and OMZ data, only the drug induced input would need to be changed for DCF and OMZ conditions, and that the reaction network could be kept exactly the same as for DEM.

Since DCF is thought not to affect Keap1 directly, we implemented an additional reaction to describe the production of ROSRM via DCF which can then modify Keap1. As a first approach we only recalibrated the parameters describing the DCF induced input using the DCF datasets ("dose_RM_sc_DCF", "degradRM_DCF", "prodROSRM", "degradROSRM", "changeKeap1_ROSRM"), while setting the other parameters of the model according to the global optimum found in the previous section. Not all data could be described using the model by this approach, especially the Keap1 mRNA had mismatches. Specifically, there the Srxn1 response in the 1000 μM DCF condition was below the 500 μM DCF response, which cannot be explained by the current model. As a second approach we jointly fitted DEM and DCF data during a combined parameter calibration, allowing also the non-DCF specific parameters to be modified in order to best describe both DCF and DEM datasets. The results for the DCF fit improved slightly compared to the first approach without deteriorating the DEM results. Finally, the DCF data was fitted standalone, allowing all parameters to adjust. This allowed for a good description of the data, yet the model was heavily overfitted, because no knockdown data was available for fitting. The fit results for all three options are presented in Fig. 4 together with the DCF data. We selected the second option, corresponding to the combined fit of the DEM and DCF datasets, for presentation in the main document.

To test the model based on DCF (i.e., with the extension of ROSRM production that can modify Keap1) on the OMZ data, as a first step we calibrated that model with only the OMZ dataset, while allowing only OMZ-related parameters and dataset-related parameters to be optimized (Fig. 6, blue). Second, we compared this with a fit where all parameters were calibrated, which significantly improved the fit (Fig. 6, red). Specifically, the log of the likelihood ratio test (logLRT) statistic was 3461 (p<0.00001), and information criteria were also consistent with acceptance of the model with the highest number of free parameters (ΔAIC: 3394; ΔBIC: 3226). While comparing the fits, we found that this was primarily due to changes in a few parameters, amongst which the Nrf2-dependent production rate of SRXN1 mRNA and the degradation rate of Srxn1-GFP. We then performed a final calibration while only allowing optimization of the OMZ-related, data-related, Nrf2-dependent SRXN1 mRNA production rate and Srxn1-GFP degradation rate, which was indeed sufficient to obtain a close fit (Fig. 6, black; Fig. 7). Also in this case the fit was significantly better than the fit where those three parameters were fixed to prior determined values

for DEM (logLRT: 3191, with $p < 0.00001$; ΔAIC:3185; ΔBIC: 3170). This suggests that the regulation by Nrf2 of SRXN1 induction is different for OMZ than for DEM and DCF.

2 Disassociation Model

For the disassociation model we incorporated a different mechanism for the RM interaction with the Keap1Nrf2 complex into the model. The modified Keap1 complex cannot bind Nrf2 anymore and the already present complex is broken again when RM attaches to it. For this setup the two-step binding of Nrf2 to Keap1 of the HL model is reduced to a single reaction since the intermediate state is not needed. In addition, eq. 5 is removed. The modified parts compared to the HL model, eqs. 1-7 and 35-42 from Table 1, are given in Table 7. The other part of the model is not changed.

References

1. Weibel, E. R., Stäubli, W., Gnägi, H. R. & Hess, F. A. Correlated morphometric and biochemical studies on the liver cell. *The Journal of Cell Biology* 68–91 (1996).
2. Eskelinen, E.-L. Macroautophagy in mammalian cells. In *Lysosomes*, 166–180 (Springer, 2005).

	Educt	→	Product	Rate
1	Nrf2 + Keap1	→	Keap1Nrf2	buildKeap1Nrf2 · Keap1 · Nrf2
2	Keap1Nrf2	→	Keap1Nrf2L	build2Keap1Nrf2 · Keap1Nrf2
3	Keap1Nrf2L	→	Keap1	decayKeap1Nrf2 · Keap1Nrf2L
4	Keap1 + P62	→	Keap1P62	buildKeap1P62 · Keap1 · P62
5	P62 + Keap1Nrf2	→	Keap1P62	buildKeap1P62H · Keap1Nrf2 · P62
6	Keap1P62	→	aKeap1P62	importKeapP62 · Keap1P62
7	aKeap1P62	→		degradKeap1P62 · aKeap1P62
8		→	Nrf2	(buildNrf2Base · (1 - Ko_Nrf2) + buildNrf2Base_Ko · Ko_Nrf2)
9	Nrf2	→	nNrf2	importNrf2 · Nrf2
10	nNrf2	→	Nrf2	exportNrf2 · nNrf2
11		→	mKeap1	buildmKeap1Base
12		→	mKeap1	buildmKeap1 · $\text{nNrf2}^{\text{hill\_Keap1}}$ / ($\text{K\_Keap1}^{\text{hill\_Keap1}}$ + $\text{nNrf2}^{\text{hill\_Keap1}}$)
13	mKeap1	→		degradmKeap1 · mKeap1
14		→	Keap1	(buildKeap1 · (1 - Ko_Keap1) + buildKeap1_Ko · Ko_Keap1) · mKeap1
15	Keap1	→		degradKeap1 · Keap1
16		→	mP62	buildmP62Base
17		→	mP62	buildmP62 · $\text{nNrf2}^{\text{hill\_P62}}$ / ($\text{K\_P62}^{\text{hill\_P62}}$ + $\text{nNrf2}^{\text{hill\_P62}}$)
18	mP62	→		degradmP62 · mP62
19		→	P62	(buildP62 · (1 - Ko_P62) + buildP62_Ko · Ko_P62) · mP62
20	P62	→		degradP62 · P62
21		→	mSrxn1	buildmSrxn1Base
22		→	mSrxn1	buildmSrxn1 · $\text{nNrf2}^{\text{hill\_Srxn1}}$ / ($\text{K\_Srxn1}^{\text{hill\_Srxn1}}$ + $\text{nNrf2}^{\text{hill\_Srxn1}}$)
23	mSrxn1	→		degradmSrxn1 · mSrxn1
24		→	Srxn1	buildSrxn1 · mSrxn1
25	Srxn1	→		degradSrxn1 · Srxn1
26		→	Gcl	buildGclBase
27		→	Gcl	buildGcl · $\text{nNrf2}^{\text{hill\_Gcl}}$ / ($\text{K\_Gcl}^{\text{hill\_Gcl}}$ + $\text{nNrf2}^{\text{hill\_Gcl}}$)
28	Gcl	→		degradGcl · Gcl
29		→	Gsh	buildGsh · Gcl
30	Gsh	→		degradGsh · Gsh
31		→	RM	2 · DRUG · (dose_RM · exp(- $(\text{time} - 72)^2$ / (2 · 2^2)) / (2 · 2.506628))
32		→	ROS	2 · DRUG · (dose_ROS · exp(- $(\text{time} - 72)^2$ / (2 · 2^2)) / (2 · 2.506628))
33	Gsh + RM	→		degradRM · RM · Gsh
34	ROS	→		degradROS · ROS
35	Keap1 + RM	→	Keap1Mod	changeKeap1_RM · Keap1 · RM
36	Keap1Nrf2 + RM	→	Keap1Nrf2Mod	changeKeap1_RM · Keap1Nrf2 · RM
37	Keap1 + ROS	→	Keap1Mod	changeKeap1_ROS · Keap1 · ROS
38	Keap1Nrf2 + ROS	→	Keap1Nrf2Mod	changeKeap1_ROS · Keap1Nrf2 · ROS
39	Keap1Mod	→		degradKeap1 · Keap1Mod
40	Nrf2 + Keap1Mod	→	Keap1Nrf2Mod	buildKeap1Nrf2 · Nrf2 · Keap1Mod
41	P62 + Keap1Mod	→	Keap1P62	buildKeap1P62 · Keap1Mod · P62
42	P62 + Keap1Nrf2Mod	→	Keap1P62	buildKeap1P62H · Keap1Nrf2Mod · P62

Table 1. Table of reactions of the full hinge-latch model.

$\frac{d}{dt}$Nrf2	=	- 1 · (buildKeap1Nrf2 · Keap1 · Nrf2) + 1 · ((buildNrf2Base · (1 - Ko_Nrf2) + buildNrf2Base_Ko · Ko_Nrf2)) - 1 · (importNrf2 · Nrf2) + 1 · (exportNrf2 · nNrf2) - 1 · (buildKeap1Nrf2 · Nrf2 · Keap1Mod)
$\frac{d}{dt}$nNrf2	=	1 · (importNrf2 · Nrf2) - 1 · (exportNrf2 · nNrf2)
$\frac{d}{dt}$Keap1	=	- 1 · (buildKeap1Nrf2 · Keap1 · Nrf2) + 1 · (decayKeap1Nrf2 · Keap1Nrf2L) - 1 · (buildKeap1P62 · Keap1 · P62) + 1 · ((buildKeap1 · (1 - Ko_Keap1) + buildKeap1_Ko · Ko_Keap1) · mKeap1) - 1 · (degradKeap1 · Keap1) - 1 · (changeKeap1_RM · Keap1 · RM) - 1 · (changeKeap1_ROS · Keap1 · ROS)
$\frac{d}{dt}$P62	=	- 1 · (buildKeap1P62 · Keap1 · P62) - 1 · (buildKeap1P62H · Keap1Nrf2 · P62) + 1 · ((buildP62 · (1 - Ko_P62) + buildP62_Ko · Ko_P62) · mP62) - 1 · (degradP62 · P62) - 1 · (buildKeap1P62 · Keap1Mod · P62) - 1 · (buildKeap1P62H · Keap1Nrf2Mod · P62)
$\frac{d}{dt}$Keap1Nrf2	=	1 · (buildKeap1Nrf2 · Keap1 · Nrf2) - 1 · (build2Keap1Nrf2 · Keap1Nrf2) - 1 · (buildKeap1P62H · Keap1Nrf2 · P62) - 1 · (changeKeap1_RM · Keap1Nrf2 · RM) - 1 · (changeKeap1_ROS · Keap1Nrf2 · ROS)
$\frac{d}{dt}$Keap1Nrf2L	=	1 · (build2Keap1Nrf2 · Keap1Nrf2) - 1 · (decayKeap1Nrf2 · Keap1Nrf2L)
$\frac{d}{dt}$Keap1P62	=	1 · (buildKeap1P62 · Keap1 · P62) + 1 · (buildKeap1P62H · Keap1Nrf2 · P62) - 1 · (importKeapP62 · Keap1P62) + 1 · (buildKeap1P62 · Keap1Mod · P62) + 1 · (buildKeap1P62H · Keap1Nrf2Mod · P62)
$\frac{d}{dt}$aKeap1P62	=	1 · (importKeapP62 · Keap1P62) - 1 · (degradKeap1P62 · aKeap1P62)
$\frac{d}{dt}$Gcl	=	1 · (buildGclBase) + 1 · (buildGcl · $nNrf2^{hill\_Gcl}$ / ($K\_Gcl^{hill\_Gcl}$ + $nNrf2^{hill\_Gcl}$)) - 1 · (degradGcl · Gcl)
$\frac{d}{dt}$Gsh	=	1 · (buildGsh · Gcl) - 1 · (degradGsh · Gsh) - 1 · (degradRM · RM · Gsh)
$\frac{d}{dt}$mSrxn1	=	1 · (buildmSrxn1Base) + 1 · (buildmSrxn1 · $nNrf2^{hill\_Srxn1}$ / ($K\_Srxn1^{hill\_Srxn1}$ + $nNrf2^{hill\_Srxn1}$)) - 1 · (degradmSrxn1 · mSrxn1)
$\frac{d}{dt}$Srxn1	=	1 · (buildSrxn1 · mSrxn1) - 1 · (degradSrxn1 · Srxn1)
$\frac{d}{dt}$mKeap1	=	1 · (buildmKeap1Base) + 1 · (buildmKeap1 · $nNrf2^{hill\_Keap1}$ / ($K\_Keap1^{hill\_Keap1}$ + $nNrf2^{hill\_Keap1}$)) - 1 · (degradmKeap1 · mKeap1)
$\frac{d}{dt}$mP62	=	1 · (buildmP62Base) + 1 · (buildmP62 · $nNrf2^{hill\_P62}$ / ($K\_P62^{hill\_P62}$ + $nNrf2^{hill\_P62}$)) - 1 · (degradmP62 · mP62)
$\frac{d}{dt}$RM	=	1 · (2 · DRUG · (dose_RM · exp(- $(time - 72)^2$ / (2 · 2^2)) / (2 · 2.506628))) - 1 · (degradRM · RM · Gsh) - 1 · (changeKeap1_RM · Keap1 · RM) - 1 · (changeKeap1_RM · Keap1Nrf2 · RM)
$\frac{d}{dt}$ROS	=	1 · (2 · DRUG · (dose_ROS · exp(- $(time - 72)^2$ / (2 · 2^2)) / (2 · 2.506628))) - 1 · (degradROS · ROS) - 1 · (changeKeap1_ROS · Keap1 · ROS) - 1 · (changeKeap1_ROS · Keap1Nrf2 · ROS)
$\frac{d}{dt}$Keap1Mod	=	1 · (changeKeap1_RM · Keap1 · RM) + 1 · (changeKeap1_ROS · Keap1 · ROS) - 1 · (degradKeap1 · Keap1Mod) - 1 · (buildKeap1Nrf2 · Nrf2 · Keap1Mod) - 1 · (buildKeap1P62 · Keap1Mod · P62)
$\frac{d}{dt}$Keap1Nrf2Mod	=	1 · (changeKeap1_RM · Keap1Nrf2 · RM) + 1 · (changeKeap1_ROS · Keap1Nrf2 · ROS) + 1 · (buildKeap1Nrf2 · Nrf2 · Keap1Mod) - 1 · (buildKeap1P62H · Keap1Nrf2Mod · P62)

Table 2. List of differential equations of the full HL model.

observable		relation with internal states
Nrf2_obs	=	log(s_nNrf2 · nNrf2 + off_nNrf2)
Keap1P62_obs	=	log(s_aKeap1P62 · aKeap1P62 + off_aKeap1P62)
Srxn1_obs	=	log(s_Srxn1 · Srxn1 + off_Srxn1)
GSH_obs	=	log(s_Gsh · Gsh + off_Gsh)
SRXN1_obs	=	log(s_mSrxn1 · mSrxn1)
SQSTM1_obs	=	log(s_mP62 · mP62)
KEAP1_obs	=	log(s_mKeap1 · mKeap1)
NFE2L2_obs	=	log(const_NFE2L2)

Table 3. List of observables.

parameter		substitution
Srxn1	=	buildSrxn1 · (K_Srxn1 $^{\text{hill\_Srxn1}}$ · buildmSrxn1Base + buildmSrxn1 · (Nrf2 · importNrf2 / exportNrf2)$^{\text{hill\_Srxn1}}$ + buildmSrxn1Base · (Nrf2 · importNrf2 / exportNrf2) $^{\text{hill\_Srxn1}}$) / (degradSrxn1 · degradmSrxn1 · (K_Srxn1$^{\text{hill_Srxn1}}$ + (Nrf2 · importNrf2 / exportNrf2)$^{\text{hill\_Srxn1}}$))
mSrxn1	=	(K_Srxn1$^{\text{hill_Srxn1}}$ · buildmSrxn1Base + buildmSrxn1 · (Nrf2 · importNrf2 / exportNrf2)$^{\text{hill\_Srxn1}}$ + buildmSrxn1Base · (Nrf2 · importNrf2 / exportNrf2)$^{\text{hill\_Srxn1}}$) / (degradmSrxn1 · (K_Srxn1$^{\text{hill_Srxn1}}$ + (Nrf2 · importNrf2 / exportNrf2)$^{\text{hill\_Srxn1}}$))
mKeap1	=	(K_Keap1$^{\text{hill_Keap1}}$ · buildmKeap1Base + buildmKeap1 · (Nrf2 · importNrf2 / exportNrf2)$^{\text{hill\_Keap1}}$ + buildmKeap1Base · (Nrf2 · importNrf2 / exportNrf2)$^{\text{hill\_Keap1}}$) / (degradmKeap1 · (K_Keap1$^{\text{hill_Keap1}}$ + (Nrf2 · importNrf2 / exportNrf2)$^{\text{hill\_Keap1}}$))
Gsh	=	buildGsh · (K_Gcl$^{\text{hill_Gcl}}$ · buildGclBase + buildGcl · (Nrf2 · importNrf2 / exportNrf2)$^{\text{hill\_Gcl}}$ + buildGclBase · (Nrf2 · importNrf2 / exportNrf2)$^{\text{hill\_Gcl}}$) / (degradGcl · degradGsh · (K_Gcl$^{\text{hill_Gcl}}$ + (Nrf2 · importNrf2 / exportNrf2)$^{\text{hill\_Gcl}}$))
aKeap1P62	=	P62 · (Keap1 · P62 · buildKeap1P62 · buildKeap1P62H + Keap1 · build2Keap1Nrf2 · buildKeap1P62 + buildKeap1P62H · buildNrf2Base) / (degradKeap1P62 · (P62 · buildKeap1P62H + build2Keap1Nrf2))
mP62	=	(K_P62$^{\text{hill_P62}}$ · buildmP62Base + buildmP62 · (Nrf2 · importNrf2 / exportNrf2)$^{\text{hill\_P62}}$ + buildmP62Base · (Nrf2 · importNrf2 / exportNrf2)$^{\text{hill\_P62}}$) / (degradmP62 · (K_P62$^{\text{hill_P62}}$ + (Nrf2 · importNrf2 / exportNrf2)$^{\text{hill\_P62}}$))
Gcl	=	(K_Gcl$^{\text{hill_Gcl}}$ · buildGclBase + buildGcl · (Nrf2 · importNrf2 / exportNrf2)$^{\text{hill\_Gcl}}$ + buildGclBase · (Nrf2 · importNrf2 / exportNrf2)$^{\text{hill\_Gcl}}$) / (degradGcl · (K_Gcl$^{\text{hill_Gcl}}$ + (Nrf2 · importNrf2 / exportNrf2)$^{\text{hill\_Gcl}}$))
Keap1P62	=	P62 · (Keap1 · P62 · buildKeap1P62 · buildKeap1P62H + Keap1 · build2Keap1Nrf2 · buildKeap1P62 + buildKeap1P62H · buildNrf2Base) / (importKeapP62 · (P62 · buildKeap1P62H + build2Keap1Nrf2))
Keap1Nrf2L	=	build2Keap1Nrf2 · buildNrf2Base / (decayKeap1Nrf2 · (P62 · buildKeap1P62H + build2Keap1Nrf2))
Keap1Nrf2	=	buildNrf2Base / (P62 · buildKeap1P62H + build2Keap1Nrf2)
nNrf2	=	Nrf2 · importNrf2 / exportNrf2
buildP62	=	Keap1P62 · importKeapP62 / mP62 + P62 · degradP62 / mP62
buildKeap1	=	Keap1 · P62 · buildKeap1P62 / mKeap1 + Keap1 · degradKeap1 / mKeap1 + Keap1Nrf2 · P62 · buildKeap1P62H / mKeap1
buildKeap1Nrf2	=	buildNrf2Base / (Keap1 · Nrf2)

Table 4. List of steady state constraints for the HL model.

name		value
Nrf2	=	-4.6 ± 0.52
alphaNuc	=	6.6 ± 0.51
exportNrf2	=	-4 ± 0.11
Keap1	=	-5.4 - 2.1 + 1.6
P62	=	1.8 - 0.66 + 0.47
buildNrf2Base	=	1.3 ± 0.28
buildKeap1P62H	=	-5.1 - 1.5 + 1.3
decayKeap1Nrf2	=	0.081 ± 0.25
degradKeap1P62	=	-0.81 - 0.31 + 0.43
K_Gcl	=	-0.065 ± 0.16
buildGclBase	=	3.5 ± 0.81
buildGcl	=	6.3 ± 0.48
degradGcl	=	-0.68 ± 0.49
K_Srxn1	=	0.14 ± 0.11
buildmSrxn1Base	=	-2 - 1 + 1.5
buildmSrxn1	=	0.73 - 0.57 + 1.4
degradmSrxn1	=	0.068 - 0.58 + 1.5
buildSrxn1	=	-2.9 ± 0.28
degradSrxn1	=	-5.3 ± 0.5
K_Keap1	=	-0.22 ± 0.13
buildmKeap1Base	=	-2.4 ± 0.52
buildmKeap1	=	0.051 - 0.36 + 0.41
degradmKeap1	=	-0.56 - 0.38 + 0.44
K_P62	=	0.25 ± 0.11
buildmP62Base	=	-1.3 - 0.49 + 0.89
buildmP62	=	0.42 - 0.46 + 0.85
degradmP62	=	-0.24 - 0.5 + 0.93
dose_ROS	=	5.6 - 1.9 + 2.1
degradROS	=	-0.33 ± 0.41
off_nNrf2	=	0.66 - 0.046 + 0.041
s_aKeap1P62	=	0.26 - 0.57 + 0.74
off_aKeap1P62	=	0.51 - 0.093 + 0.07
off_Gsh	=	0.94 - 0.037 + 0.031
const_NFE2L2	=	-0.059 ± 0.081
sigma_abs_Nrf2_obs	=	-2.4 ± 0.059
sigma_abs_Keap1P62_obs	=	-1.7 ± 0.057
sigma_abs_Srxn1_obs	=	-2 ± 0.061
sigma_abs_GSH_obs	=	-2.2 ± 0.11
sigma_abs_SRXN1_obs	=	-0.71 - 0.2 + 0.23
sigma_abs_SQSTM1_obs	=	-0.97 - 0.2 + 0.23
sigma_abs_KEAP1_obs	=	-0.58 - 0.22 + 0.25
sigma_abs_NFE2L2_obs	=	-1.3 - 0.2 + 0.23
degradRM_DEM	=	9.8 ± 2.1
dose_RM_sc	=	-1.7 ± 0.55
Delta_buildKeap1P62	=	2 - 1.7 + 2.1
Delta_buildNrf2Base_KO	=	0.8 ± 0.087
Delta_buildKeap1_KO	=	1 ± 0.089
Delta_buildP62_KO	=	0.5 - 0.21 + 0.16
hill_Srxn1	=	1.3 ± 0.18

Table 5. Parameter values on logarithmic scale for the best converged fit after model reduction. Uncertainties were retrieved from the parameter profiles in Fig. 2.

subset	name	treatment	dose [μM]	knockdown
DEM timecourse	DMSO_0_CTRL_timecourse	DMSO	0	CTRL
	DEM_10_CTRL_timecourse	DEM	10	CTRL
	DEM_100_CTRL_timecourse	DEM	100	CTRL
	DEM_316_CTRL_timecourse	DEM	316	CTRL
	DEM_561_CTRL_timecourse	DEM	561	CTRL
DEM knockdown	DMSO_0_CTRL_knockdown	DMSO	0	CTRL
	DMSO_0_KEAP1_knockdown	DMSO	0	KEAP1
	DMSO_0_NRF2_knockdown	DMSO	0	NRF2
	DMSO_0_p62_knockdown	DMSO	0	p62
	DEM_316_CTRL_knockdown	DEM	316	CTRL
	DEM_316_KEAP1_knockdown	DEM	316	KEAP1
	DEM_316_NRF2_knockdown	DEM	316	NRF2
	DEM_316_p62_knockdown	DEM	316	p62
DCF timecourse	DCF_100_CTRL_timecourse	DCF	100	CTRL
	DCF_316_CTRL_timecourse	DCF	316	CTRL
	DCF_500_CTRL_timecourse	DCF	500	CTRL
	DCF_1000_CTRL_timecourse	DCF	1000	CTRL
OMZ timecourse	OMZ_5_CTRL_timecourse	OMZ	5	CTRL
	OMZ_24_CTRL_timecourse	OMZ	24	CTRL
	OMZ_47_CTRL_timecourse	OMZ	47	CTRL
	OMZ_94_CTRL_timecourse	OMZ	94	CTRL
	OMZ_188_CTRL_timecourse	OMZ	188	CTRL
	OMZ_282_CTRL_timecourse	OMZ	282	CTRL

Table 6. List of datasets used for modeling. The datasets are separated into four groups: (1) the DEM time course data corresponding to time course data of cells treated with various concentrations of DEM, (2) the DEM knockdown data corresponding to experiments for knock-down of Keap1, Nrf2 and p62, (3) the DCF time course data corresponding to experiments with various concentrations of DCF, and (4) the OMZ time course data corresponding to experiments with various concentrations of OMZ. The treatment is specified in the third column, where DMSO stands for the control treatment with no drug. The drug concentration is given in the fourth column and the knock-down state in the last column.

	Educt	$\rightarrow$	Product	Rate
1b	Nrf2 + Keap1	$\rightarrow$	Keap1Nrf2	buildKeap1Nrf2 · Keap1 · Nrf2
3b	Keap1Nrf2	$\rightarrow$	Keap1	decayKeap1Nrf2 · Keap1Nrf2
4	Keap1 + P62	$\rightarrow$	Keap1P62	buildKeap1P62 · Keap1 · P62
6	Keap1P62	$\rightarrow$	aKeap1P62	importKeapP62 · Keap1P62
7	aKeap1P62	$\rightarrow$		degradKeap1P62 · aKeap1P62
35	Keap1 + RM	$\rightarrow$	Keap1Mod	changeKeap1_RM · Keap1 · RM
36b	Keap1Nrf2 + RM	$\rightarrow$	Nrf2 + Keap1Mod	changeKeap1_RM · Keap1Nrf2 · RM
37	Keap1 + ROS	$\rightarrow$	Keap1Mod	changeKeap1_ROS · Keap1 · ROS
38b	Keap1Nrf2 + ROS	$\rightarrow$	Nrf2 + Keap1Mod	changeKeap1_ROS · Keap1Nrf2 · ROS
39	Keap1Mod	$\rightarrow$		degradKeap1 · Keap1Mod
41	P62 + Keap1Mod	$\rightarrow$	Keap1P62	buildKeap1P62 · Keap1Mod · P62

Table 7. Modified reactions for the dissassociation model.

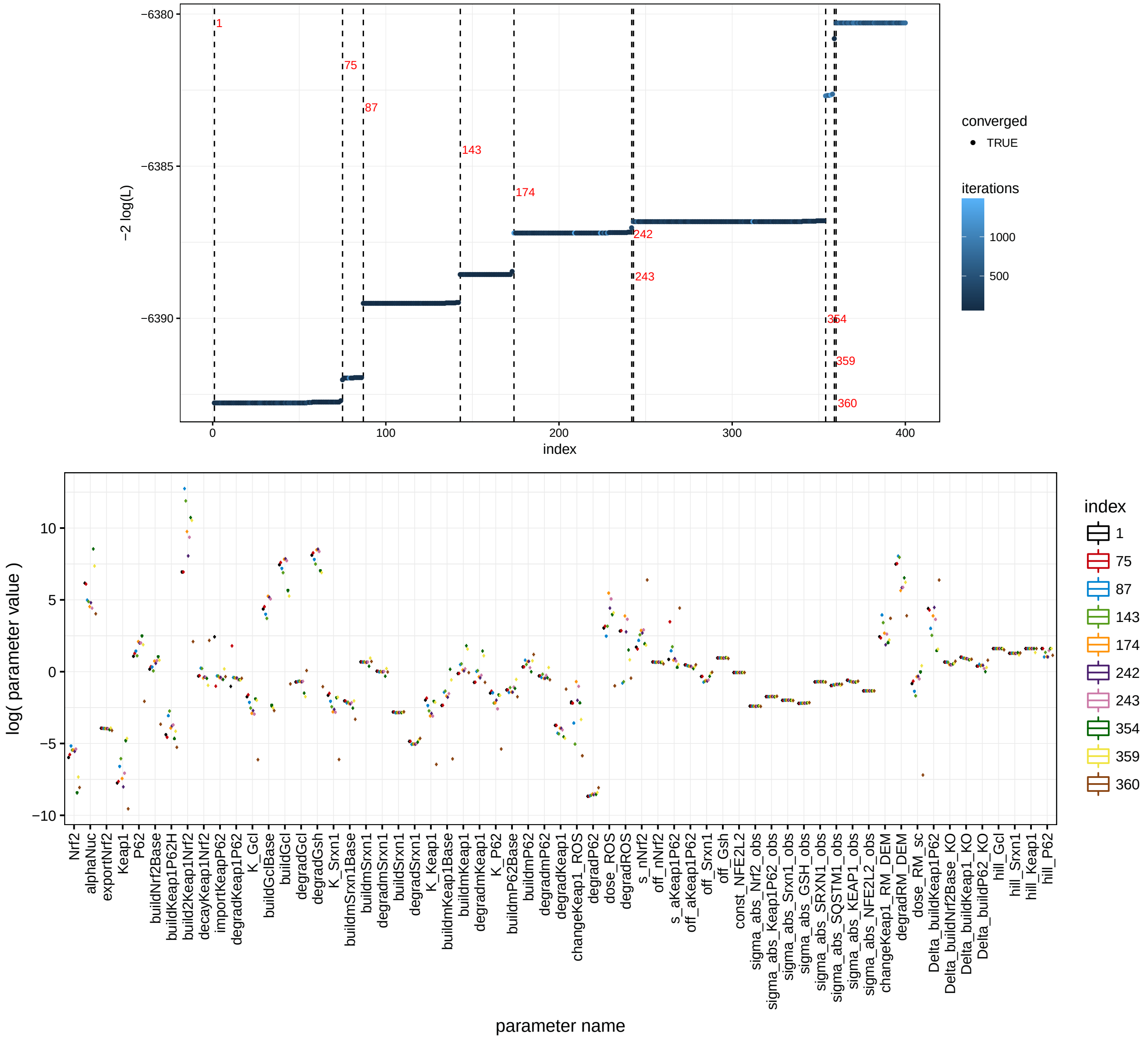

Figure 1. Top panel: Ordered values of the 400 best converged fits before applying any model reductions. Vertical lines separate the local optima with a 0.1 tolerance. Bottom panel: Corresponding parameter values for the 400 best converged fits.

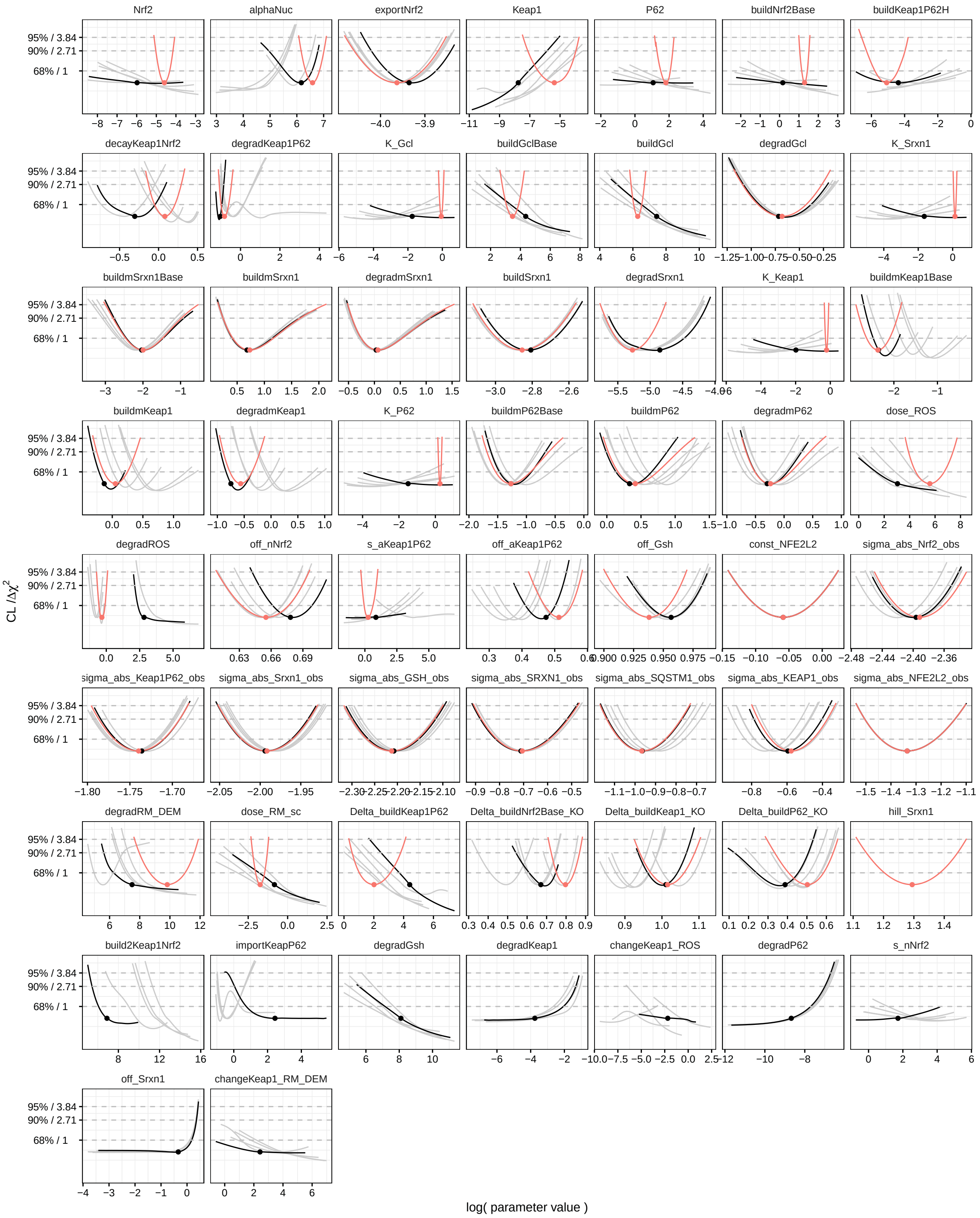

Figure 2. Parameter Profiles obtained for the HL model before applying any model reductions (black: best optimum, grey: optimum 2-5) and of the best optimum of the fully reduced model (red). For the black and grey curves only the data contribution to the objective function is included, not showing the prior contributions. No priors were added for the reduced model in red.

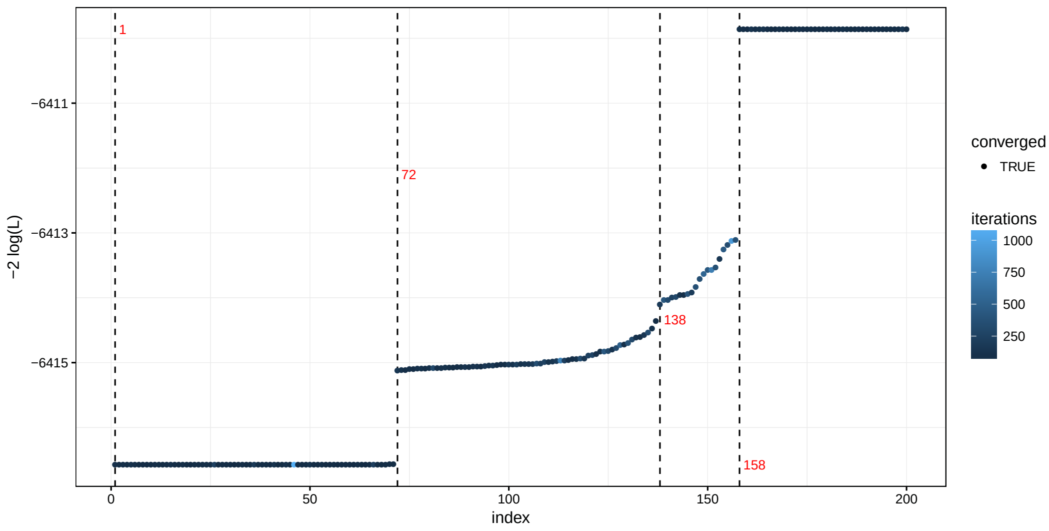

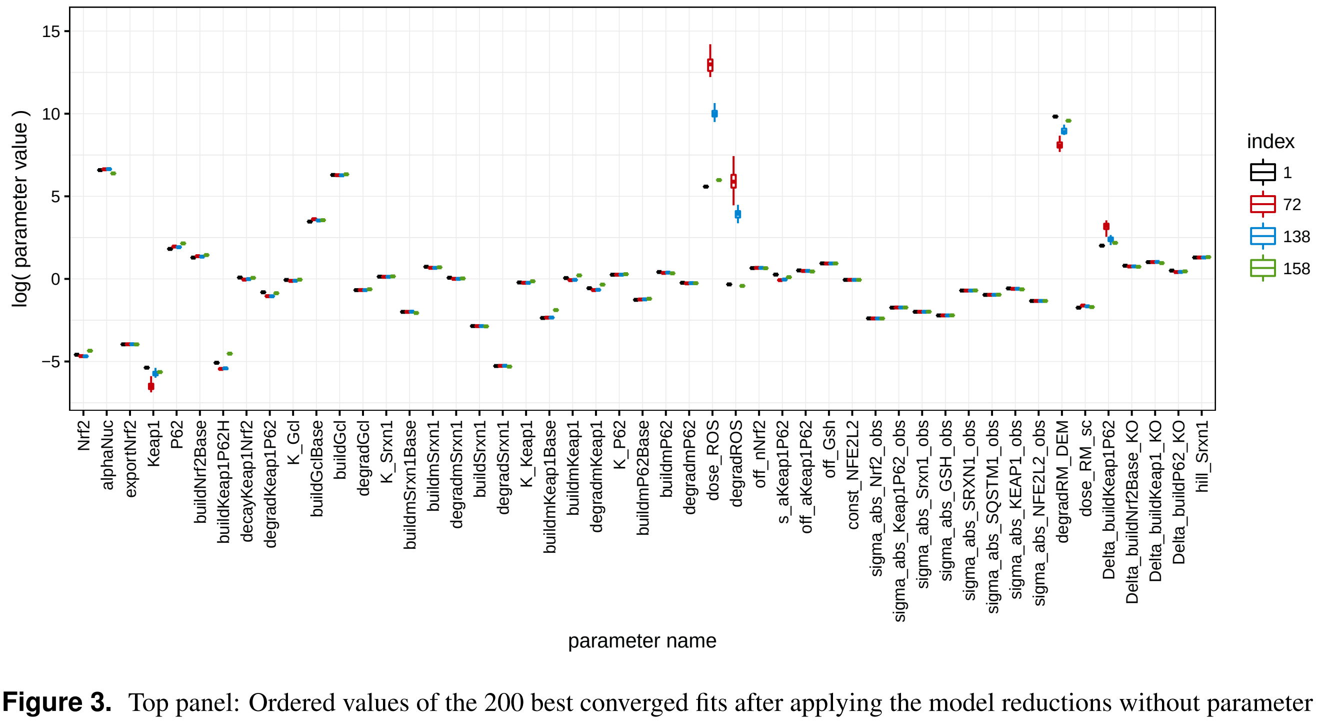

Figure 3. Top panel: Ordered values of the 200 best converged fits after applying the model reductions without parameter priors. Vertical lines separate the local optima with a 1.0 tolerance. Bottom panel: Corresponding parameter values for the 200 best converged fits.

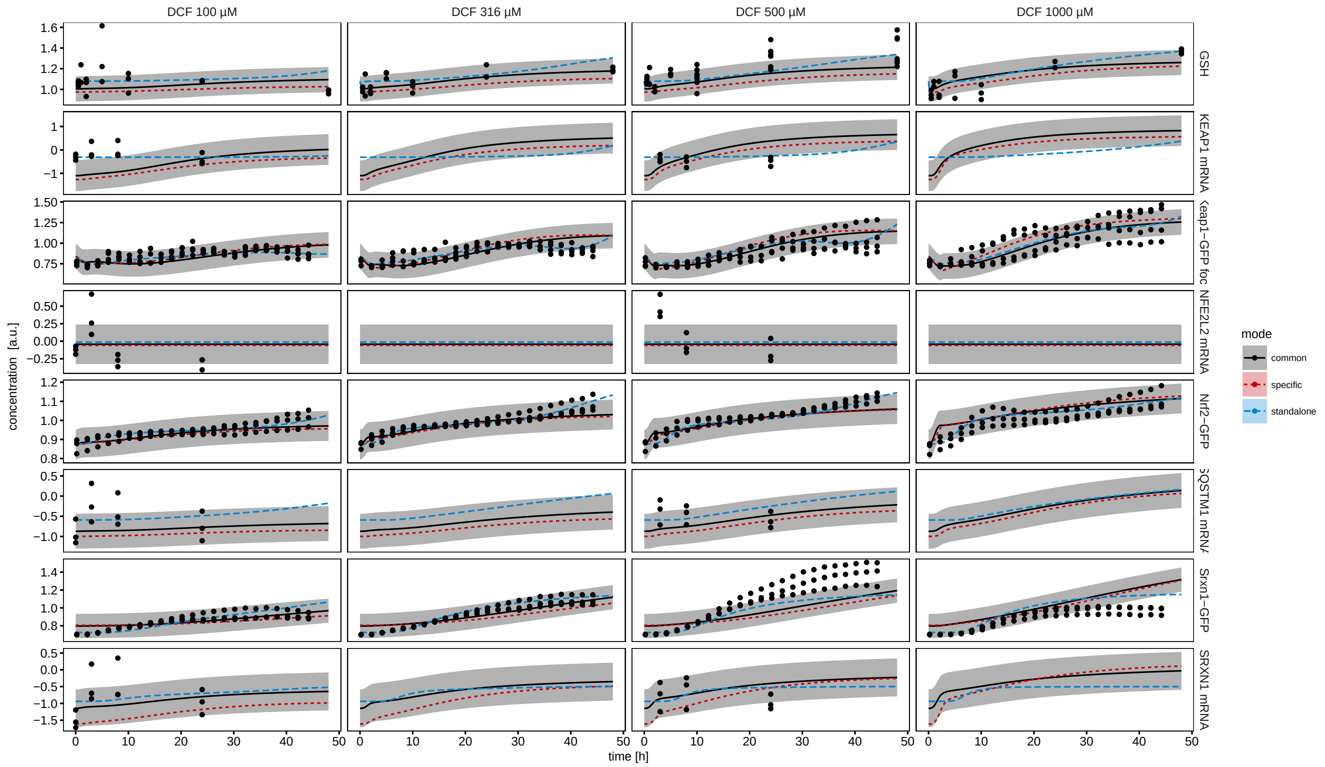

Figure 4. Fit results of the HL model for the DCF data. Either only the DCF specific parameters were adapted (red: specific), or all parameters were estimated from all datasets together in a combined fit (black: common, with uncertainty band), or from the DCF data standalone (blue: standalone).

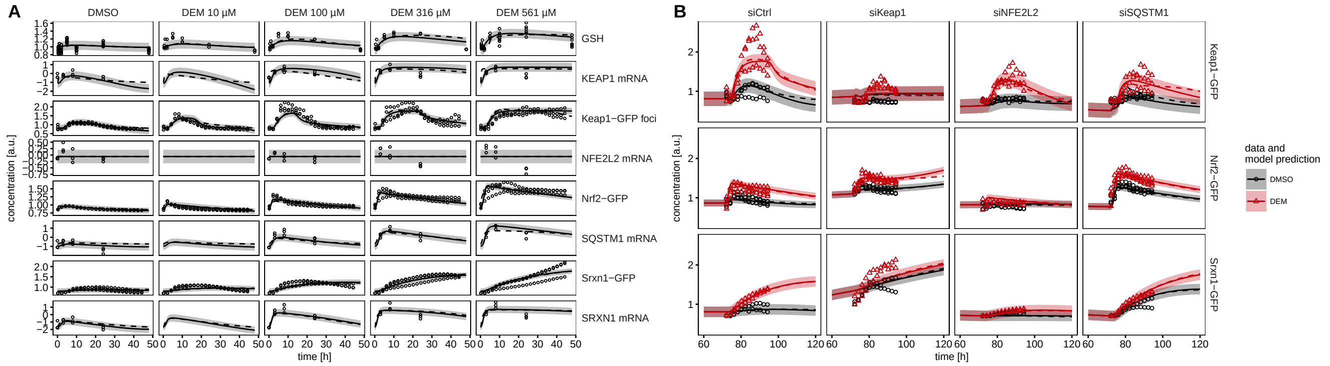

Figure 5. Fitted model trajectories and data for the disassociation model for the DEM timecourse A) and knockdown datasets B). The result for the disassociation model is given by solid lines and uncertainty bands. The result of the hinge-latch model is shown as dashed lines for comparison.

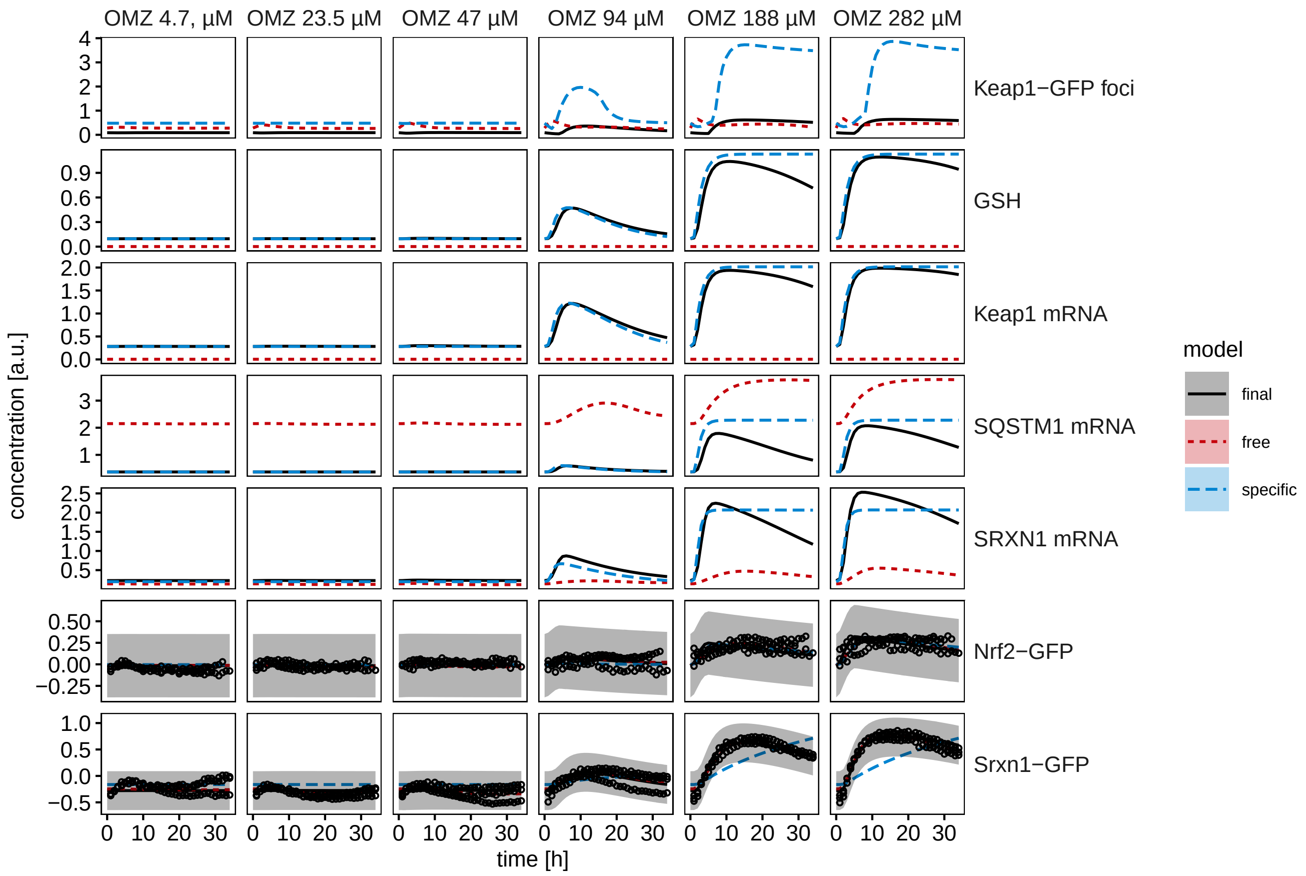

Figure 6. Fit results of the HL model for the OMZ data. Either only the OMZ specific parameters were adapted (blue: specific), or all parameters were estimated from the OMZ data (red: free), or OMZ specific parameters and Nrf2-dependent production rate of SRXN1 mRNA, and degradation of Srxn1-GFP1 were estimated (black: final, with uncertainty band).

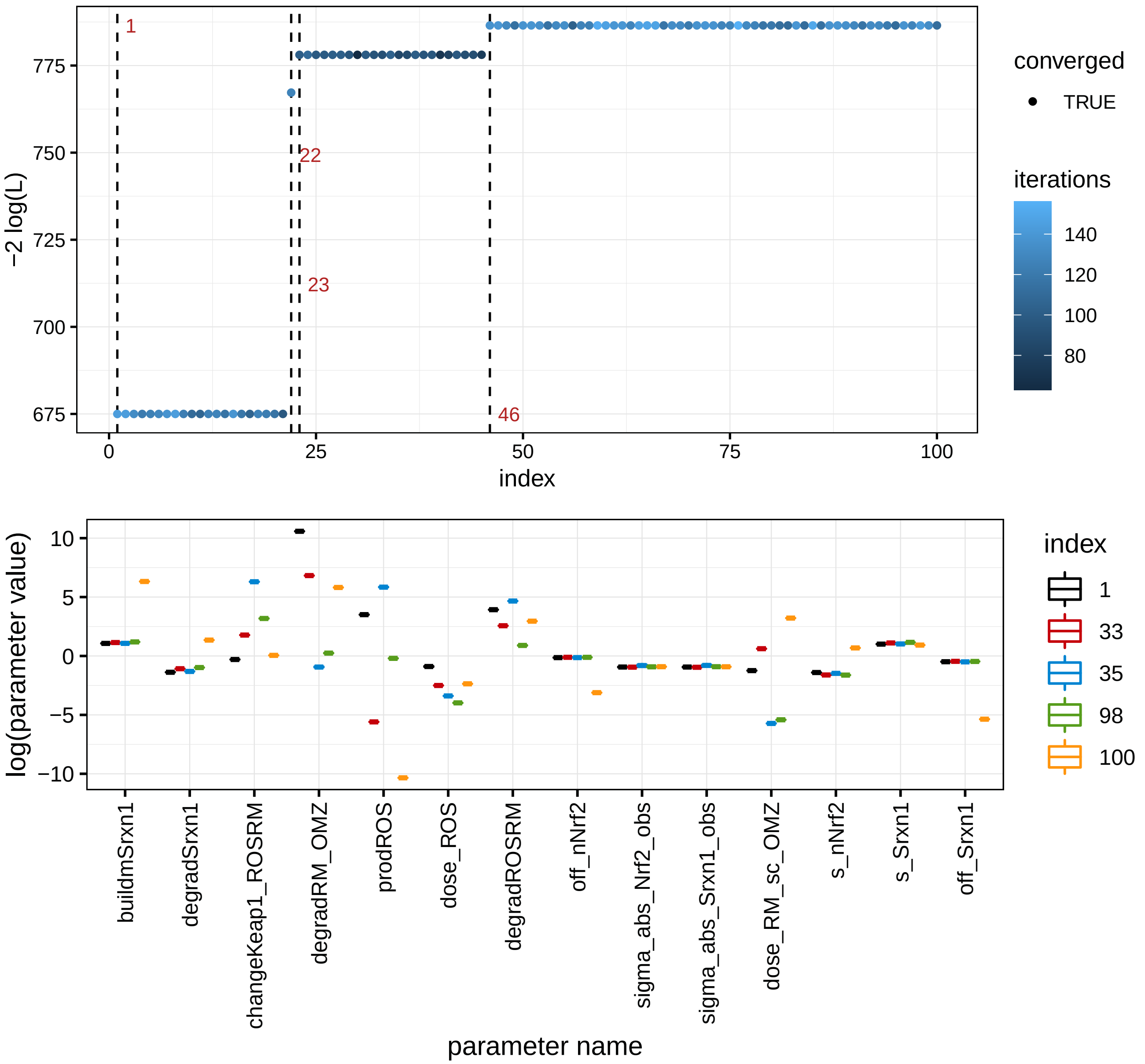

Figure 7. Top panel: Ordered values of the 100 best converged model fits to the OMZ data, for the case where the fitted parameters included OMZ related parameters, Nrf2-dependent production rate of SRXN1 mRNA, and degradation rate of Srxn1-GFP. Vertical lines separate the local optima with a 1.0 tolerance. Bottom panel: Corresponding parameter values for the 100 best converged fits.
